# New Insights in the Synthesis of High-Molecular-Weight Aromatic Polyamides-Improved Synthesis of Rod-like PPTA

**DOI:** 10.3390/ijms24032734

**Published:** 2023-02-01

**Authors:** Guiomar Hernández, Sergio Ferrero, Helmut Reinecke, Camino Bartolomé, Jesús M. Martinez-Ilarduya, Cristina Álvarez, Ángel E. Lozano

**Affiliations:** 1 Instituto de Ciencia y Tecnología de Polímeros, ICTP-CSIC, E-28006 Madrid, Spain; 2 Department of Chemistry-Ångström Laboratory, Uppsala University, SE-751 21 Uppsala, Sweden; 3 IU CINQUIMA, Universidad de Valladolid, E-47011 Valladolid, Spain; 4 Associated Research Unit to CSIC, Facultad de Ciencias, Universidad de Valladolid, E-47011 Valladolid, Spain

**Keywords:** polyamidation, in situ silylation, poly(*p*-phenylene terephthalamide) (PPTA), Kevlar, NMR, DFT molecular simulation

## Abstract

By employing a variation of the polyamidation method using in situ silylated diamines and acid chlorides, it was possible to obtain a rod-type polyamide: poly(*p*-phenylene terephthalamide) (PPTA, a polymer used in the high-value-added material Kevlar), with a molecular weight much higher than that obtained with the classical and industrial polyamidation method. The optimization of the method has consisted of using, together with the silylating agent, a mixture of pyridine and a high-pKa tertiary amine. The research was complemented by a combination of nuclear magnetic resonance and molecular simulation studies, which determined that the improvements in molecular weight derive mainly from the formation of silylamide groups in the growing polymer.

## 1. Introduction

To achieve materials with improved properties for advanced applications, polymers with the highest possible molecular weight should be synthesized. During the development of macromolecular chemistry, many synthetic methods have been optimized to obtain the total conversion of reactive groups, to use mild conditions, to limit the risk of side secondary reactions, to increase the easiness of operation, etc.

Concerning the polycondensation reactions able to produce wholly aromatic polyamides (commonly named aramids), the most common method both in industry and academia consists of the reaction between diacid chlorides and aromatic diamines in aprotic polar solvents at low temperatures [1,2,3,4,5,6]. However, it is necessary to add Ia and/or IIa salts to improve the solubility of the aromatic polyamide when seeking to obtain high-molecular-weight polymers, particularly when wholly rod-like aramids are the polymer target [3,7,8,9]. Another synthetic approach (when acid chlorides are difficult to attain or sometimes when the low-temperature polyamidation method is not well-suited to the presence of reactive groups, e.g., hydroxyl moieties) consists of the reaction of aromatic diamines and aromatic diacids at temperatures between 80 and 120 °C, employing phosphorous derivatives as promoters (Yamazaki–Higashi method) [3,10,11,12,13].

A milestone in the synthesis of aromatic polyamides was the use of silylated diamines in the 1980s. These nucleophilic monomers are more reactive and produce higher-molecular-weight (Mw) polyamides. This methodology started with research carried out by Imai when working with diverse polymers, polyamides [14,15], and other polyheterocycles [16] and also was employed for other macromolecular structures [17,18,19]. Afterward, our group developed a new synthetic method, following a described method for poly (amic ethyl ester)s [20] by using silylated diamines formed in situ and obtaining poly (isophthalamides) with higher Mw values than those obtained using the reported methods [21,22].

Later, we applied the in situ silylation method to obtain other polymer structures, and the results were also excellent and better than those observed using classical polyamidation methodologies. In particular, the method was applied to the synthesis of aromatic polyimides [23,24], where the need to stoichiometrically add a base such as pyridine (Py) was shown, supplemented with a small amount of a higher-pKa base, such as 4-dimethylaminopyridine (DMAP), to obtain very-high-molecular-weight polyimides, clearly higher than those observed when other methodologies were employed [23,24]. This polyimide synthesis methodology was later employed successfully for making high-molecular-weight polymers such as poly(o-hydroxyimide)s and poly(o-hydroxyamide)s, i.e., precursors of thermally rearranged polybenzoxazoles (TR-PBOs) [25,26,27,28,29].

On the other hand, rod-like polymers, also called extended polymers, are a type of material with very interesting properties due to their macromolecular topology, especially for their use as high-strength and high-modulus materials [8,9,30,31,32]. Rod-like polymers are built from various macromolecular structures, so the required synthetic methodology is broad [3,33]. For rod-type aramids, the industry usually carries out their synthesis by employing low-temperature polyamidation and using high-torque stirrers and aprotic polar solvents mixed with 5–10% lithium or calcium salts. Regarding the Yamazaki–Higashi synthesis, the use of this high-temperature methodology was employed for high-Mw rod-like polyamides, and specifically, this methodology gave the aromatic polyamide derived from *p*-phenylenediamine and terephthalic acid, namely poly(*p*-phenylene terephthalamide), (commonly named as PPTA or PPD-T) [34,35], with the highest inherent viscosity ever reported for this rod-like polymer (higher than 9 dL/g) [36].

When using silylated diamines in polyamidation, it is noteworthy to comment that Imai [15] found that the gelation point for the PPTA synthesis was achieved using longer times than using conventional classical techniques. However, no further investigations were carried out on this finding although this result seemed to point to a different polymerization mechanism for PPTA.

**Scheme 1:**
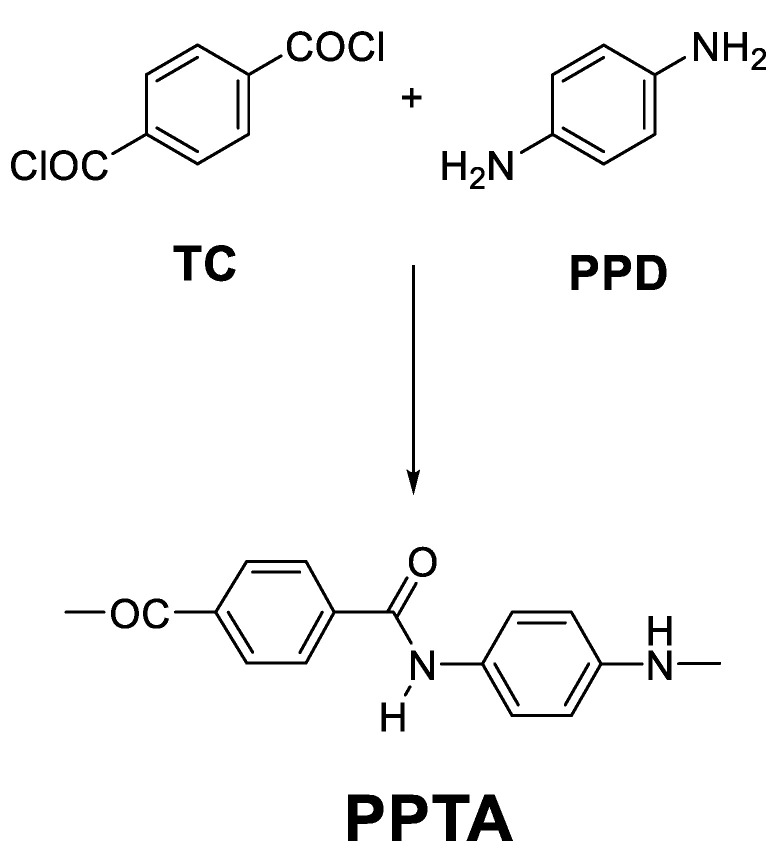
Synthesis of PPTA from terephthaloyl chloride (TC) and *p*-phenylene diamine (PPD).

With all these results in mind, this communication deals mainly with the synthesis of polyamide PPTA (Scheme 1) by using three different methods: (1) classical low-temperature polyamidation method between diacid chlorides and diamines, (2) Yamazaki–Higashi polyamidation between diacids and diamines, and (3) a base-assisted in situ silylation polyamidation method between diacid chlorides and diamines, as proposed in this work.

The Yamazaki–Higashi polyamidation reaction, employing diacids and diamines and triphenyl phosphite as a reaction promoter at 115 °C, is used for comparison purposes.

In this work, the in situ silylation of PPD, by using trimethylsilyl chloride (TMSC), is explored in the formation of PPTA, where pyridine (Py) is used as a promoter base for the reaction together with small amounts of a high-pKa tertiary amine such as 4-dimethylaminopyridine (DMAc).

In addition, to test the usefulness of the proposed modification for the in situ silylation polyamidation reaction by the addition of tertiary bases, the study is extended to more soluble polyamides, namely polyisophthalamides, where aromatic diamines with different electronic properties are used, and a diamine possessing steric groups in ortho position is also employed to observe the differences in the molecular weights of the polymers produced by the different methods.

Finally, to give a plausible explanation for the results obtained in this work, a DFT computational study was carried out together with an experimental study by nuclear magnetic resonance (NMR).

In conclusion, this work aims to find a simple and easy synthetic method to obtain aromatic rod-like polyamides, particularly PPTA, of high Mw.

## 2. Results and Discussion

### 2.1. Optimization of the Polyamidation Reaction

In order to obtain aromatic polyisophthalamides, the in situ silylation of aromatic diamines was employed. (Details about the synthesis of polyisophthalamides are shown in Supporting Information, Section S1.) The isophthalic acid-derived aramids are much more soluble than rod-type aramids, and therefore, existing or non-existing improvements in molecular weight could be more easily detected. In Table S1 of the Supporting Information section, the inherent viscosity values of polyisophthalamides denoted that the use of the modified in situ silylation of aromatic diamines greatly enlarged the Mw of these polymers. Thus, 3.5-1.7-fold improvements in inherent viscosity values were observed. The largest improvement was observed for diamine 6FpDA (4,4′ (hexafluoroisopropylidene) dianiline), with relatively low reactivity, and the lowest one for the diamine DDS (4,4′-sulfonyldianiline), which has a very strong electron-withdrawing sulfone group. For sterically hindered diamines, such as 4M, the improvement when using TMSC and Py was 2.6 fold, much greater than that observed for the reaction employing TMSC and no base (1.2 fold).

In any case, the η_inh_ increments were large enough to conclude that this modified polyamidation method is excellent and produces polyisophthalamides with degrees of polymerization far superior to those obtained by non-silylation, classical low-temperature polyamidation.

As a preliminary conclusion, when making polyisophthalamides, the correct choice of synthesis would be to employ the in situ silylation method without pyridine except when hindered aromatic diamines are to be used, where the addition of pyridine produces a considerable improvement in their degree of polymerization. Due to these findings, we considered that this method could be applied to the synthesis of rod-like aromatic polyamides such as PPTA.

Thus, samples of PPTA obtained in five batches under different reaction conditions of in situ silylation were compared with those prepared by the classical PPTA polymerization and the Yamazaki–Higashi phosphorous-assisted reaction. (Details about this methodology are shown in the Supporting Information, Section S2.) The inherent viscosity data included in Table 1 permitted us to assert that much-higher-molecular-weight PPTAs were obtained by the silylated-promoted method. In particular, the use of TMSC and pyridine, in a stoichiometric relationship (batch reaction 2), increased the η_inh_ value from 3.15 to 4.36 dL/g. This inherent viscosity improvement is important since, according to the Mark–Houwink–Sakurada equation of PPTA (Mw = 3902.4η^1.556^) [37,38], the polymerization degree was roughly 65% higher. However, when a small amount of DMAP was employed together with pyridine (batch reaction 3, 20% DMAP/Py), the inherent viscosity value jumped to a value of 6.7 dL/g (3 times higher Mw than that having 3.15 dL/g). The use of larger amounts of TMSC or DMAP (batch reactions 4 and 5) produced high η_inh_ polymers but slightly lower than the PPTA made from a stoichiometric relationship between Py and the amine groups of PPD of 2 and a mixture DMAP/Py ratio of 20% (batch reaction 3). Molecular weights of PPTAs estimated from Mark–Houwink–Sakurada equation are listed in Table S6 in the Supporting Information, Section S6.

The η_inh_ values of PPTAs made with this in situ silylation methodology were superior to that PPTA made by us using the Yamazaki–Higashi method (6.1 dL/g). At this point, we should remark that all reactions were carried out using a simple mechanical stirrer at 100–150 rpm with a common Teflon stirrer blade.

The time to reach the gel point was also approximately determined. The critical gel point of PPTA by employing the modified in situ polyamidation method was reached in longer times (1.5–2 fold) than that obtained by the classical low-temperature reaction, as was observed by Imai [15].

In conclusion, we developed a simple polyamidation methodology able to produce PPTA from common monomers (TC and PPD) having very high Mw, which is comparable to that of PPTA made by the industry.

Following this finding, an attempt was made to explain the observed improvement in the synthesis of PPTA. At first glance, the differences in reactivity appeared to us to be inconsistent in explaining the improvement of the reaction. Therefore, it was assumed that either enhancement in the solubility of the rod-like polymer or changes in its chain conformation could explain these differences. Therefore, we assumed that a certain amount of amide groups formed during the reaction could be silylated.

Thus, to validate our assumption, a molecular modeling study using mechano-quantum methods as well as an NMR study was accomplished.

### 2.2. Theoretical and NMR Study of PPTA Synthesis

In the first step, as was done in previous works [24,39], the DFT (B3LYP/6–31G(d,p)), HOMO, and LUMO energies of acid chlorides and aromatic diamines were compared. It is well-known that the reactivity of the amidation reaction depends on the ΔEgap = E_HOMO_nucleophile (diamine)-E_LUMO_electrophile (acid chloride), so the smaller the energy difference between those orbitals, the higher its reactivity. The reactivity scale, determined by the ΔEgap, was preserved for both unsilylated and silylated diamines, as observed in Table S3 (Supporting Information, Section S3).

On the other hand, concerning the synthesis of PPTA synthesis, it was observed that the PPD/TC monomer pair should be highly reactive because the energy gap E_HOMO_ (PPD)-E_LUMO_ (TC) is small, particularly smaller than other monomer pairs that yield high-molar-weight polyamides such as, for example, isophthalic chloride (IC) and m-phenylene diamine (MPD) (Table 2 and Table S3 of Supporting Information).

**Scheme 2:**
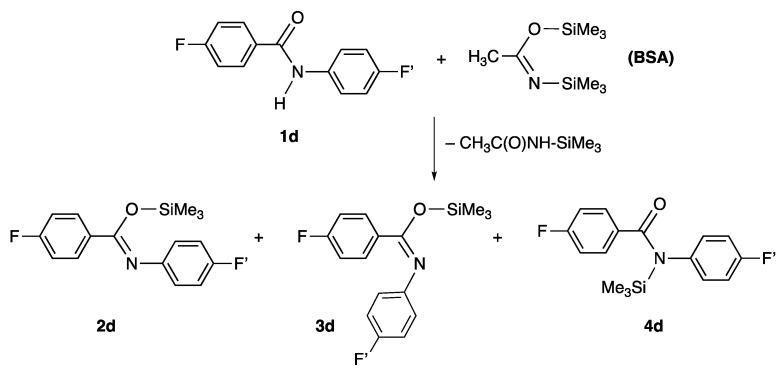
Silylation of 4-fluoro-N-(4-fluorophenyl)benzamide **1d** by using the silylating agent BSA.

**Figure 1:**
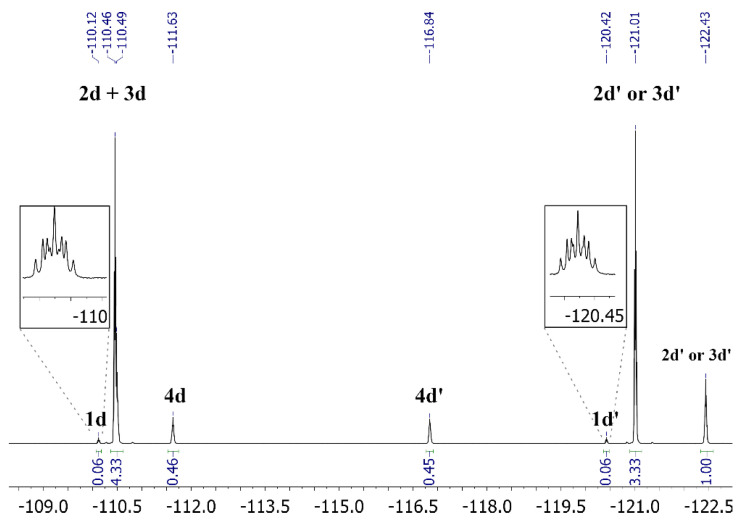
^19^F NMR (470 MHz, acetone-d_6_ cap.) spectrum at 283 K from the silylation reaction of **1d** with N,O-bis (trimethylsilyl) acetamide (BSA) in DMAc. The quotation mark (′) depicts the fluorine atom on the right in the structures shown in Scheme 2.

The NMR study performed on a set of selected benzanilides (Scheme S2 of the Supporting Information, Section S4.2) allowed us to determine that the most stable silylated amide isomers corresponded to the *trans* and *cis* O-silyl imidate species 2 and 3. As an example, Figure 1 shows the ^19^F NMR spectrum of the reaction mixture of the fluorinated benzamides obtained from silylation of 4-fluoro-N-(4-fluorophenyl)benzamide using N,O-bis (trimethylsilyl) acetamide (BSA) as silylating agent (Scheme 2). All of the details about this study are provided in the Supporting Information, Section S4.2.

The isomer stability scale determined by DFT (B3LYP/6-31G(d,p)) molecular modeling turned out to be completely the same as that determined in the NMR study for the silylated benzanilide of Scheme S3 (Supporting Information, Section S5.2).

It should be noted that this *cis* O-silyl imidate isomer has a completely different conformation from that of the N-silyl amide (silylated benzanilide 4, Scheme S3) and the *trans* O-silyl imidate isomer (silylated benzanilide 2, Scheme S3).

With the structures optimized by DFT calculations, macromolecular structures of PPTA were created using the MaterialsStudio package [40].

**Figure 2:**
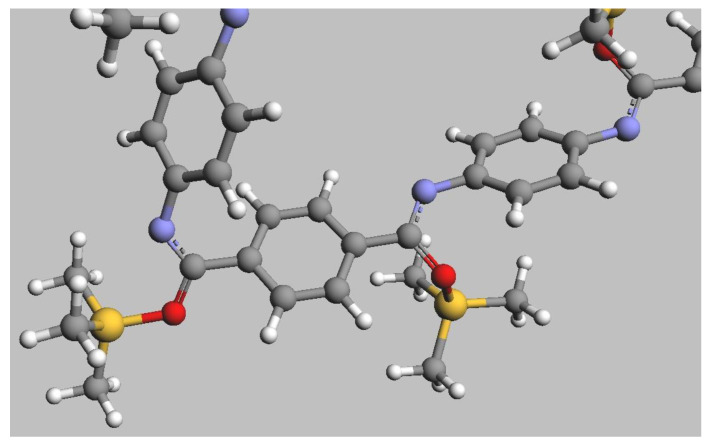
Molecular representation of a PPTA chain in which the amide groups are silylated, with one of them having a *cis* conformation (left part of the graph) and the other a *trans* conformation (right part of the graph).

For the macromolecular models derived from *trans* O-silyl imidate 2 and N-silyl amide 4, the macromolecular structures were observed to be extended. However, in the case of the *cis* O-silyl imidate 3, the macromolecular structure was a not-extended chain (Figure 2 and Figure S14 in Supporting Information, Section S5.2).

Therefore, the improvement in the degree of polymerization observed for the reactions carried out using the modified in situ silylation method could be justified for two reasons: (*i*) The presence of bulky trimethylsilyl groups in the PPTA chains determines both a worse interchain packing and a lower amount of hydrogen bonds (due to a larger interchain distance), which results in a higher solubility of the polymer, and consequently, the progress of the polyamidation reaction is more favorable since the PPTA precipitation occurs at longer times. (*ii*) In the macromolecular chains, there must be a certain amount of *cis* O-silyl imidate groups (*cis*-type amides) formed during the polyamidation process, which produce a fold (*kink*) in the extended chain. Therefore, the presence of this type of conformation should produce an improvement in solubility, and therefore, as in the previous case, precipitation of the polymer will be observed at longer times, along with a greater increase in molecular weight.

This finding, the increase of the molecular weight of a polymer due to an improvement of its solubility in the polymerization medium, is well-accepted. For example, the Higashi–Yamazaki PPTA reaction formation, which takes place at high temperatures and gives very-high-molecular-weight polyamides, could be seen in this sense, where the high temperature employed (>105 °C) and the interactions of the amide groups with the phosphorus compounds could enhance the solubility of the growing polymer.

## 3. Materials and Methods

Terephthaloyl chloride (TC) and isophthaloyl chloride (IC) (TCl Europe, Zwijndrecht, Belgium, and Sigma-Aldrich, Madrid, Spain, respectively) were recrystallized twice from hexane and subsequently sublimed at high vacuum just before use. High-purity 1,4-phenylene diamine (PPD, Sigma-Aldrich, Madrid, Spain) was sublimed under vacuum before being used. Trimethylsilyl chloride (TMSC), anhydrous pyridine (Py), and anhydrous N-methyl-2-pyrrolidinone (NMP) were purchased from Merck (Madrid, Spain) and used as received. High-purity 1,3-phenylene diamine (MPD); 4,4′-oxidianiline (ODA); 4,4′-(hexafluoroisopropylidene) dianiline (6FpDA); and 4,4′-methylene-bis(2,6-dimethylaniline) (4M) were all purchased from Merck (Madrid Spain), and they were sublimed just before use. 4,4′-Sulfonyldianiline (Merck, Madrid, Spain) (DDS) was recrystallized from water and dried at 120 °C under vacuum prior to use. N,N-Dimethylaminopyridine (DMAP) was purchased from TCI, Zwijndrecht, Belgium. Terephthalic acid (TA, Amoco, Chicago, Illinois, U.S.) was used after drying under vacuum at 100 °C. Calcium and lithium chloride (Scharlau, Barcelona, Spain) were dried at 250 °C overnight and kept in a desiccator with phosphorous pentoxide under vacuum. Triphenyl phosphite (TPP, Merck, Madrid, Spain) was distilled under vacuum and kept in a desiccator with phosphorous pentoxide under a low vacuum. Other materials and solvents were commercially available and used as received.

### 3.1. Characterization

Inherent viscosities of all polyisophthalamides (η_inh_) were determined in 0.5 g/dL NMP solutions at 30 °C in a Cannon Ubbelohde viscometer placed in a Lauda iVisc device. Inherent viscosities of PPTA (η_inh_) were determined in 0.1 g/dL 98% H_2_SO_4_ solutions at 30 °C in a Cannon Ubbelohde viscometer placed in a Lauda iVisc device.

Computer simulations were carried out by first drawing the molecules in GaussView 5 [41] and then optimizing the structures at the AM1 level [42]. Subsequently, electronic energies of the optimized geometries were calculated by density functional theory (DFT), without any geometrical constraint (use of Opt keyword) for starting molecules and final molecules, using the Becke’s three-parameter hybrid function with the 6–31G(d,p) basis set B3LYP/6–31G(d,p) [43,44], employing the Gaussian 09 program [45,46]. Molecular depictions were created using the Arguslab 4.01 freeware program [47] and the GaussView 5 program [41].

The NMR methodology employed in this work is described in the Supporting Information (Section S4.1 NMR methods).

### 3.2. Synthesis of Poly (p-Phenylene Terephthalamide) (PPTA) Using the Yamazaki–Higashi Method

For the synthesis of PPTA using the Yamazaki–Higashi polyamidation, a modification of the method proposed by Mariani was employed [12,13,36] by heating the mixture of terephthalic acid and *p*-phenylene diamine at 115 °C during 12 h in the presence of triphenyl phosphite and pyridine as promoters (for synthetic details, see Supporting Information, Section S2).

### 3.3. Synthesis of Poly (p-Phenylene Terephthalamide) (PPTA) Using the Classical Methodology between TC and PPD

All polyamidation reactions were carried out employing a PTFE stirrer blade (Sigma-Aldrich, Madrid, Spain) and a mechanical non-high-torque stirrer device (IKA).

For the classical synthesis of PPTA, the polymerization reaction was accomplished as follows: a double-walled glass flask was charged with an amount of aromatic diamine (PPD, 5 mmol), calcium chloride (2 g), and NMP (25 mL) under a blanket of nitrogen. After the diamine dissolved, the stirred solution was cooled to 0 °C, and a stoichiometric amount of diacid chloride (TC, 5 mmol) was added. The mixture was allowed to react under nitrogen for 1 h at 5 °C and then for 12 h at 20 °C.

### 3.4. Synthesis of Poly (p-Phenylene Terephthalamide) (PPTA) Using the In Situ Silylation Method

For the synthesis of PPTA, four experimental setups were arranged for the PPD and TC pair. Each flask, equipped with a mechanical stirrer (speed of 150 rpm) and under a nitrogen atmosphere, was charged with 15 mL of NMP, calcium chloride (2 g), and 5.0 mmol of diamine. Each mixture was stirred at room temperature until all solids had dissolved. Then, the solutions were cooled to 0 °C, and the required amount of TMSC (10.0 mmol) was slowly added to the second flask, the necessary amount of TMSC and Py (10.0 mmol of each) was added to the third one, and the needed amounts of Py (10.0 mmol) and DMAP (1.0 mmol) were added to the fourth flask. Subsequently, the temperature of all flasks was raised to room temperature, and the solutions were stirred for 15 min to ensure the formation of the silylated diamine in the appropriate flasks (flasks 2, 3, and 4). After this time, the solutions were once more cooled to 0 °C, and TC (5.0 mmol) was rapidly added, followed by 15 mL of NMP to each flask. The reaction mixtures were stirred for 15 min at 0 °C, and then, the temperature was left to increase to room temperature and then left overnight. Afterward, the resulting polymer gel was precipitated into 200 mL of water, chopped in a blender, and washed several times with water/ethanol 1/1 and ethanol to remove traces of solvent and oligomers. All the polymers were dried overnight in an oven under vacuum at 120 °C. Yields over 98% were obtained.

## 4. Conclusions

The methodology of in situ silylation of diamines in the synthesis of aromatic polyamides has been extended to the synthesis of a high-molecular-weight rod-like poly (*p*-phenylene terephthalamide), namely PPTA, by adding to the silylation solution pyridine together with a non-stochiometric amount (0.2–0.4 mol/mol of Py) of a high-pKa tertiary diamine (N,N-dimethylaminopyridine).

The use of this methodology has allowed for obtaining PPTA with an inherent viscosity value of 6.7 dL/g similar to, if not higher than, that of commercial PPTA. It seems clear that following this research, the use of more specific equipment for the synthesis of rod-like materials (e.g., the use of a high-shear twin screw stirrer with a blade) should improve the final molecular weight to some extent.

Based on Imai’s observation [15] that the apparent gelation time of PPTA increased when silylated diamines were used, it was thought that one of the main reasons for the observed improvement in the molecular weight of PPTA might be due to a higher solubility of the growing polyamide chains in solution because a certain amount of the amide groups were silylated.

This idea was justified based on NMR and molecular modeling studies using model benzamides. It was found that the conformations of silylated PPTA chains could favor their solubility due to a worse packing of the chains.

This simple and easy-to-apply approach could be extended to other types of rod-like or very rigid polyamides (for instance, poly(p-benzamide)s, PBAs, and copoly(*p*-phenylene/3,4′-diphenylether terephthalamide)) [32] made by the reaction of a diacid chloride with an aromatic diamine.

## Figures and Tables

**Table 1:** Inherent viscosities (η_inh_, dL/g) of PPTA as a function of silylating agent, base (Py), and cobase (DMAP).

Batch Reaction	TMSC ^a^	TMSC /Py ^b^	TMSC/Py/DMAP ^c^	η_inh_ ^d,e^
1	-	-	-	3.2 ± 0.1
2	2	2	-	4.4 ± 0.1
3	2	2	20	6.7 ± 0.1
4	2	4	20	6.3 ± 0.1
5	2	4	40	6.6 ± 0.1

^a^ 2 mol TMSC/1 mol PPD; ^b^ x mol Py/1 mol PPD; ^c^ (y mol% DMAP/x mol Py); ^d^ measured in 98% H_2_SO_4_ solutions at 30 ± 0.1 °C, with 0.1 g/dL polymer concentration; ^e^ standard deviations from repeated measurements.

**Table 2:** HOMO energy values (eV) of PPD and silylated PPD. Energy gap (eV) of the reaction between PPD and terephthaloyl chloride (TC).

	E_HOMO_ (eV)	Energy Gap (eV) * E_HOMO_[PPD]–E_LUMO_[TC]
PPD	−4.75	−1.84
Silylated PPD	−4.41	−1.50

* Orbital frontier energy gap of the reaction of PPD with TC. E_LUMO_ energy of TC = −2.91 eV.

## Data Availability

Not applicable.

## Supplementary Materials

The following supporting information can be downloaded at: https://www.mdpi.com/article/10.3390/ijms24032734/s1.
