# Supplementary material for: New Insights in the Synthesis of High-Molecular-Weight Aromatic Polyamides-Improved Synthesis of Rod-like PPTA
Source: Int J Mol Sci. 2023 Feb 1;24(3):2734. doi: 10.3390/ijms24032734 (PMC9917457; doi:10.3390/ijms24032734)
Supplement: Supplementary file 1 [file ijms-24-02734-s001.zip › ijms-2115408-supplementary.pdf]

Supporting Information

New insights in the synthesis of high molecular weight aromatic polyamides.

Improved synthesis of rod-like PPTA

Guiomar Hernández[1,2], Sergio Ferrero[3], Helmut Reinecke[1], Camino Bartolomé[3], Jesús M. Martinez-Ilarduya[3,*], Cristina Alvarez[1,3,4], Angel E. Lozano[1,3,4,*]

1. Instituto de Ciencia y Tecnología de Polímeros, ICTP-CSIC, Juan de la Cierva 3, E-28006 Madrid, Spain
2. Department of Chemistry – Ångström Laboratory, Uppsala University, Box 538, SE-751 21 Uppsala, Sweden
3. IU CINQUIMA, Universidad de Valladolid, Paseo Belén 5, E-47011 Valladolid, Spain
4. SMAP, UA-UVA_CSIC, Associated Research Unit to CSIC. Universidad de Valladolid, Facultad de Ciencias, Paseo Belén7, E-47011 Valladolid, Spain

* corresponding authors; jmi@qi.uva.es and lozano@ictp.csic.es

S1. Synthesis of polyisophthalamides

S2. Synthesis of poly(p-phenylene terephthalamide) (PPTA) using the Yamazaki-Higashi method

S3. Electronic parameters of monomers

S4. NMR study of the silylation of benzanilide models

S4.1 NMR methods

S4.2 Experimental discussion

S5. Molecular simulation study

S5.1. Molecular simulation methods

S5.2. Molecular simulation discussion

S6. Molecular weight of PPTAs.

S7. Additional information

S1. Synthesis of polyisophthalamides

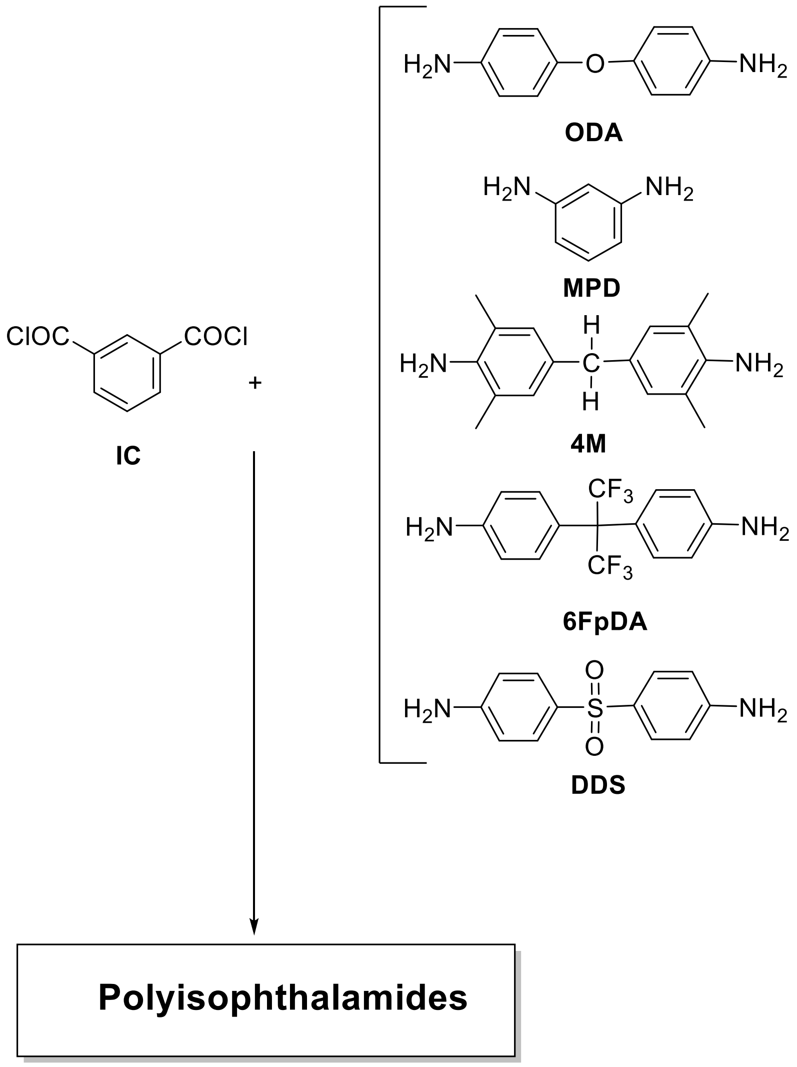

For the synthesis of polyisophthalamides, three experimental setups were arranged for each diamine/IC pair. Each flask, equipped with a mechanical stirrer under a nitrogen atmosphere was charged with 5 mL of anhydrous N-methyl-2-pyrrolidinone (NMP) and 5.0 mmol of diamine. Each mixture was stirred at room temperature until all solids had dissolved. Then, the solutions were cooled to 0 °C and the required amount of trimethylsilyl chloride (TMSC) (10.0 mmol) was slowly added to the second flask, and the necessary amount of TMSC and anhydrous pyridine (Py) (10.0 mmol of each) was added to the third flask. The temperature of the three flasks was raised to room temperature, and the solutions were stirred for 15 min to ensure the formation of the silylated diamine in the appropriate cases. After this time, the solutions were once more cooled to 0 °C, and IC (5.0 mmol) was rapidly added followed by 5 mL of NMP. The reaction mixtures

were stirred for 15 min at 0 °C and then the temperature was let to raise up to room temperature and left overnight. Afterward, the resulting polymer solutions were precipitated into 200 mL of water, and washed several times with water/ethanol 1/1 to remove traces of solvent and oligomers. All the polymers were dried overnight under vacuum at 120 °C. Yields over 95% were obtained.

Table S1. Inherent viscosities (η_{inh}, dL/g) of polyisophthalamides measured at 30 ºC using NMP as solvent.

Diamine	η_{inh}^{0} (a)	η_{inh}^{TMSC} (b)	$\eta_{inh}^{TMSC+Py}$ (c)	$\eta_{inh}^{TMSC}/\eta_{inh}^{0}$	$\eta_{inh}^{TMSC+Py}/\eta_{inh}^{0}$
ODA	0.90	2.54	2.27	2.8	2.5
MPD	0.8	2.10	1.85	3.5	2.8
4M	0.25	0.31	0.65	1.2	2.6
6FpDA	0.75	2.37	2.23	3.2	3.0
DDS	0.58	1.02	0.99	1.8	1.7

(a) **Flask 1**: IC: 5 mmol, diamine: 5 mmol and NMP: 10 mL; (b) **Flask 2**: IC: 5 mmol, diamine: 5 mmol, NMP: 10 mL and TMSC: 10 mmol; (c) **Flask 3**: IC: 5 mmol, diamine: 5 mmol, NMP: 10 ml, TMSC: 10 mmol and Py: 10 mmol. Inherent viscosity values having a 2% error.

S2. <u>Synthesis of</u> poly(p-phenylene terephthalamide) (PPTA) using the Yamazaki-Higashi method.

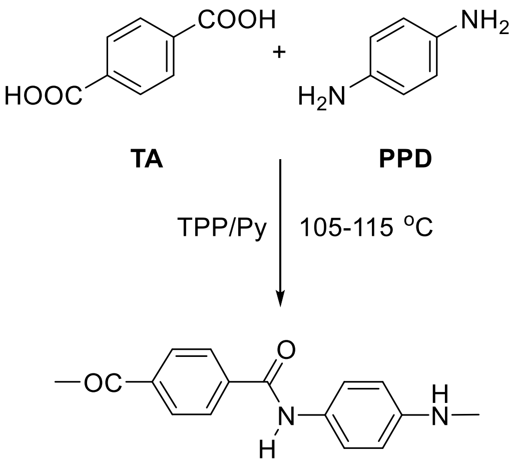

For the synthesis of **PPTA** using the Yamazaki-Higashi polyamidation a modification of the method proposed by Mariani was employed[1]. A 250 mL flask, equipped with a reflux condenser and a mechanical stirrer (speed of 150 rpm) under a nitrogen atmosphere was charged with 15 mL of NMP, calcium chloride (2 g), and 5.0 mmol of PPD. The mixture was stirred at room temperature until all solid got dissolved. Then, the solution was placed in a silicone oil bath to room temperature and the required amount of TA powder (5.0 mmol) and triphenyl phosphite (TPP) (10 mmol) was added into the flask, and the reaction mixture was stirred for 5 min., slowly heated to 115 °C and held at that temperature for 12 h with stirring under a nitrogen flow. Afterward, the resulting polymer gel was precipitated into 200 mL of water, washed in hot water twice, ground in a blender, and washed several times consecutively with hot water, water/ethanol 1/1, and ethanol to remove traces of solvent and promotor compounds. All the polymers were dried overnight under vacuum at 120 °C. Quantitative yield was obtained. Inherent viscosity (98% H_2SO_4) of **PPTA** was 6.1 dL/g

S3. Electronic parameters of monomers

Table S2. LUMO energy of aromatic diacid chlorides

Diacid chloride	E_{LUMO} (eV)
IC	-2.77
TC*	-2.91

* TC; terephthaloyl chloride

Table S3. HOMO energy (eV) of aromatic diamines and their corresponding silylated diamines. Energy gap (eV) of the reaction between diamines and isophthaloyl chloride

Diamine	$E_{HOMO\ [diamine]}$	$E_{HOMO[silylated\ diamine]}$	$E_{HOMO[diamine]}$-$E_{LUMO[IC]}$ gap *	$E_{HOMO[silylated\ diamine]}$-$E_{LUMO[IC]}$ gap *
Aniline	-5.39	-5.23	-2.62	-2.46
ODA	-4.86	-4.70	-2.09	-1.93
MPD	-5.07	-4.87	-2.30	-2.10
PPD	-4.75	-4.41	-1.98	-1.64
4M	-4.91	-5.08	-2.14	-2.31
6FpDA	-5.50	-5.42	-2.73	-2.65
DDS	-5.67	-5.61	-2.90	-2.84

*E_{LUMO} energy of IC=-2.77 eV

S4. NMR study of the silylation of benzanilide models

S4.1 NMR methods

NMR spectra were recorded on a 500 MHz Agilent DD2 instrument equipped with a One NMR probe or HFX probe (inverse triple resonance probe) in the Laboratory of Instrumental Techniques (LTI) Research Facilities, University of Valladolid. ^{1}H, ^{13}C, ^{29}Si and ^{15}N NMR chemical shifts (δ) are reported in parts per million (ppm) and are referenced to tetramethylsilane (TMS) or nitromethane, when corresponding. Coupling constants (J) are reported in Hz. ^{1}H, ^{13}C, ^{29}Si and ^{15}N peaks assignments were performed using 2D NMR methods (^{1}H-^{13}C HSQC, ^{1}H-^{29}Si HMBC,^{1}H-^{15}N HMBC and $^{19}F\{^{1}H\}$–^{15}N HMBC) employing adiabatic pulses in the low frequency channel. In the case of the special $^{19}F\{^{1}H\}$–^{15}N HMBC spectrum, the long-range $^{5}J_{19F\text{-}15N}$ coupling were optimized to 5 Hz. The mixing time employed in the ^{19}F-^{19}F EXSY was 200 ms. Two different temperatures, 283 and 295 K, were conducted to acquire all the multinuclear NMR studies. Quantitative $^{13}C\{^{1}H\}$ and $^{29}Si\{^{1}H\}$ NMR analyses were carried out employing the inverse-gated decoupling method, in order to obtain a proton decoupled spectrum without NOE enhancements. In addition, a small amount of paramagnetic relaxation agents (chromium (III) acetylacetonate, ($Cr(acac)_3$) were added to the NMR tube with the aim of reducing the T_1 relaxation time of ^{13}C and ^{29}Si nuclei. The acquisition parameters for quantitative measurements of the different nuclei were: ^{1}H: 5 s relaxation delay between transients, 45° pulse width, spectral width of 6510.42 Hz, a total of 1 transient and 5 s acquisition time; ^{19}F: 7 s relaxation delay between transients, 30° pulse width, spectral width of 8503.40 Hz, a total of 32 transients and 5 s acquisition time; $^{13}C\{^{1}H\}$: 1.5 s relaxation delay between transients, 80° pulse width, spectral width of 6281.41 Hz, a total of 2000 transients and 5 s acquisition time; $^{29}Si\{^{1}H\}$: 2.5 s relaxation delay between transients, 60° pulse width, spectral width of 3980.89 Hz, a total of 6000 transients and 5 s acquisition time. The obtained NMR spectra were manipulated and processed using Mestrelab Research software (MNova 12.0) (Santiago de Compostela, Spain) and VnmrJ4.2 software (Agilent). Prior to NMR analysis, all data obtained were subsequently apodized with line broadening window function and zero-filling was applied to improve the digital resolution of the final spectrum.

S4.2 Experimental discussion

a) In the first stage, the stoichiometric reaction of 4-(*tert*-butyl)benzoyl chloride and aniline was carried out in DMSO-d_6 employing N,O-bis(trimethylsilyl)acetamide (BSA) as silylating agent.

The synthetic protocol was as follows; in a 5 mL round flask magnetically stirred and N_2 blanketed, 0.5 mmol of aniline was dissolved in 0.5 mL of DMSO-d_6. After cooling the reaction at 10 ºC, 0.5 mmol of BSA (dissolved in 0.5 mL of DMSO-d_6) was dropwise added into the flask. After 5 min, a 0.5 mL aliquot was taken out, placed in a NMR tube, and characterized by ^{1}H-NMR. Into the flask, 0.25 mmol of 4-(*tert*-butyl)benzoyl chloride in 0.25 mL of DMSO-d_6 was added, stirred for 60 min, and the obtained solution was also characterized by ^{1}H-NMR.

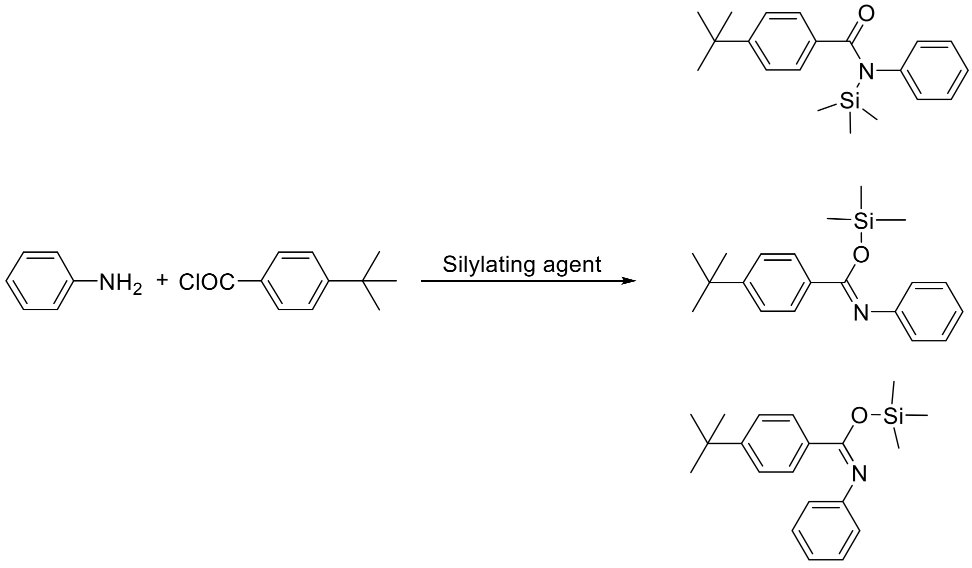

Scheme S1. Reaction of 4-(*tert*-butyl)benzoyl chloride and aniline in the presence of a silylating agent

In Figure S1, it is observed the reaction of aniline with BSA. The NMR spectra were carried out after 5 min of reaction, and it could be observed that the silylation of aniline was complete, since the ratio between the NMR peak at around 5.2 ppm and the aromatic protons was 1 to 5.

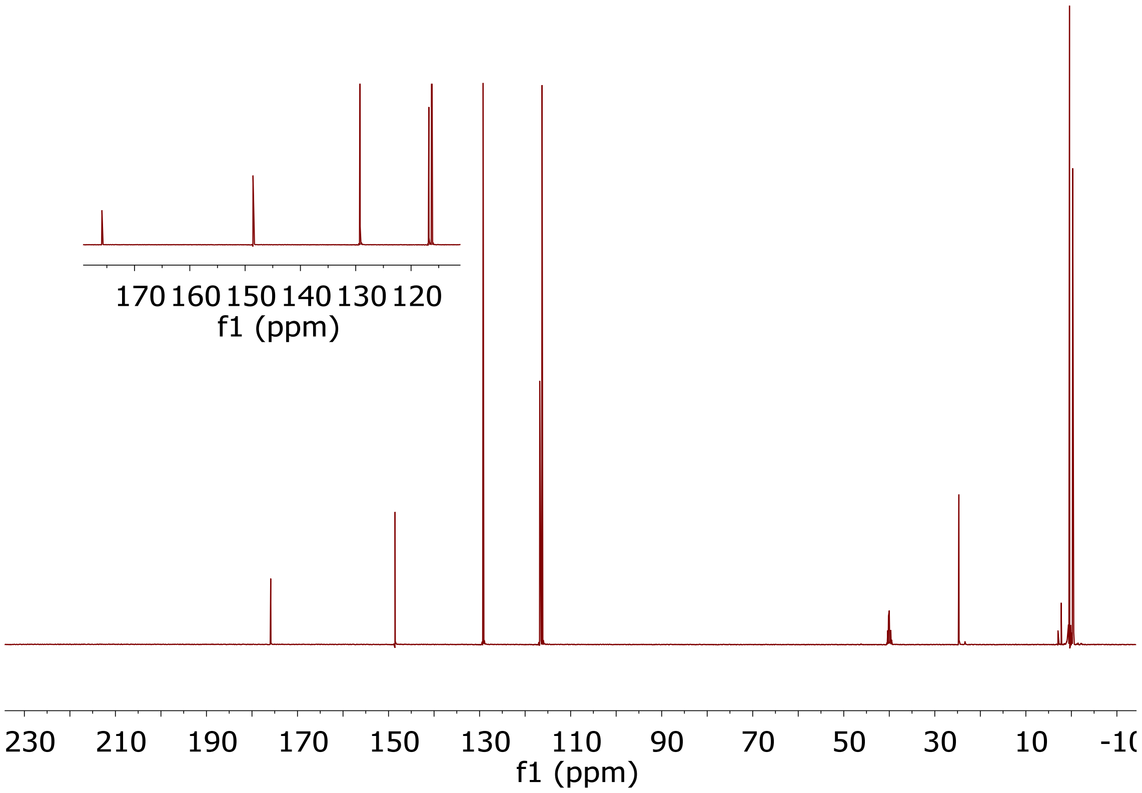

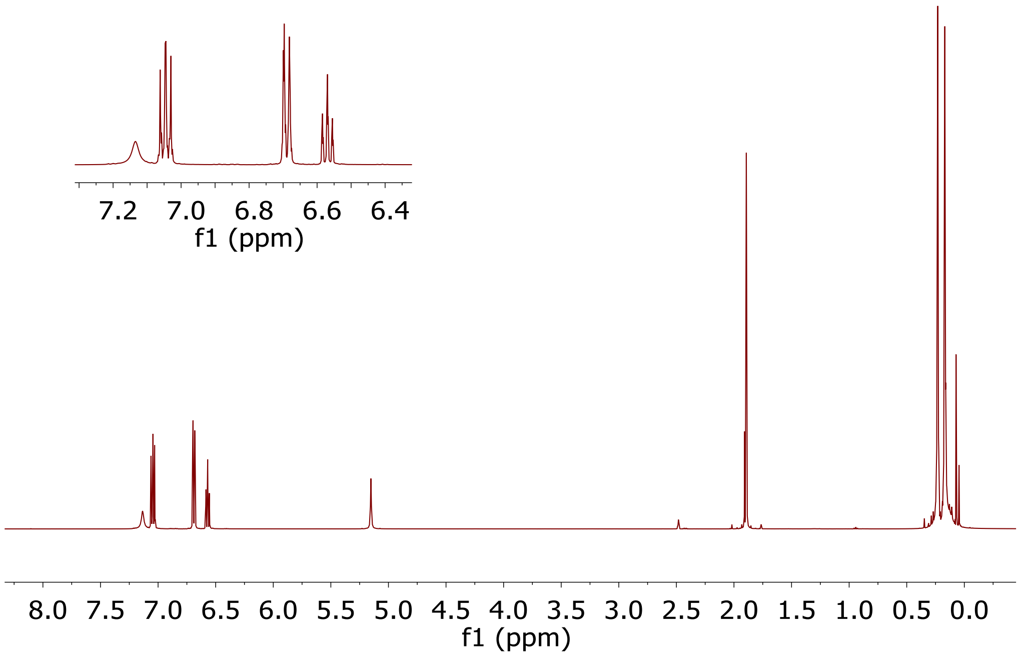

Figure S1. Reaction of aniline with BSA in DMSO-d_6. Top) $^{13}C\{^1H\}$-NMR spectrum; bottom) ^1H-NMR spectrum.

In this reaction, the predominant formation of silylated benzanilide groups was observed. However, the formation of a small amount of non-silylated benzanilide was seen (NMR doublet peak that appeared at low-field (around 8.05 ppm) in the ^1H-NMR spectrum of Figure S2).

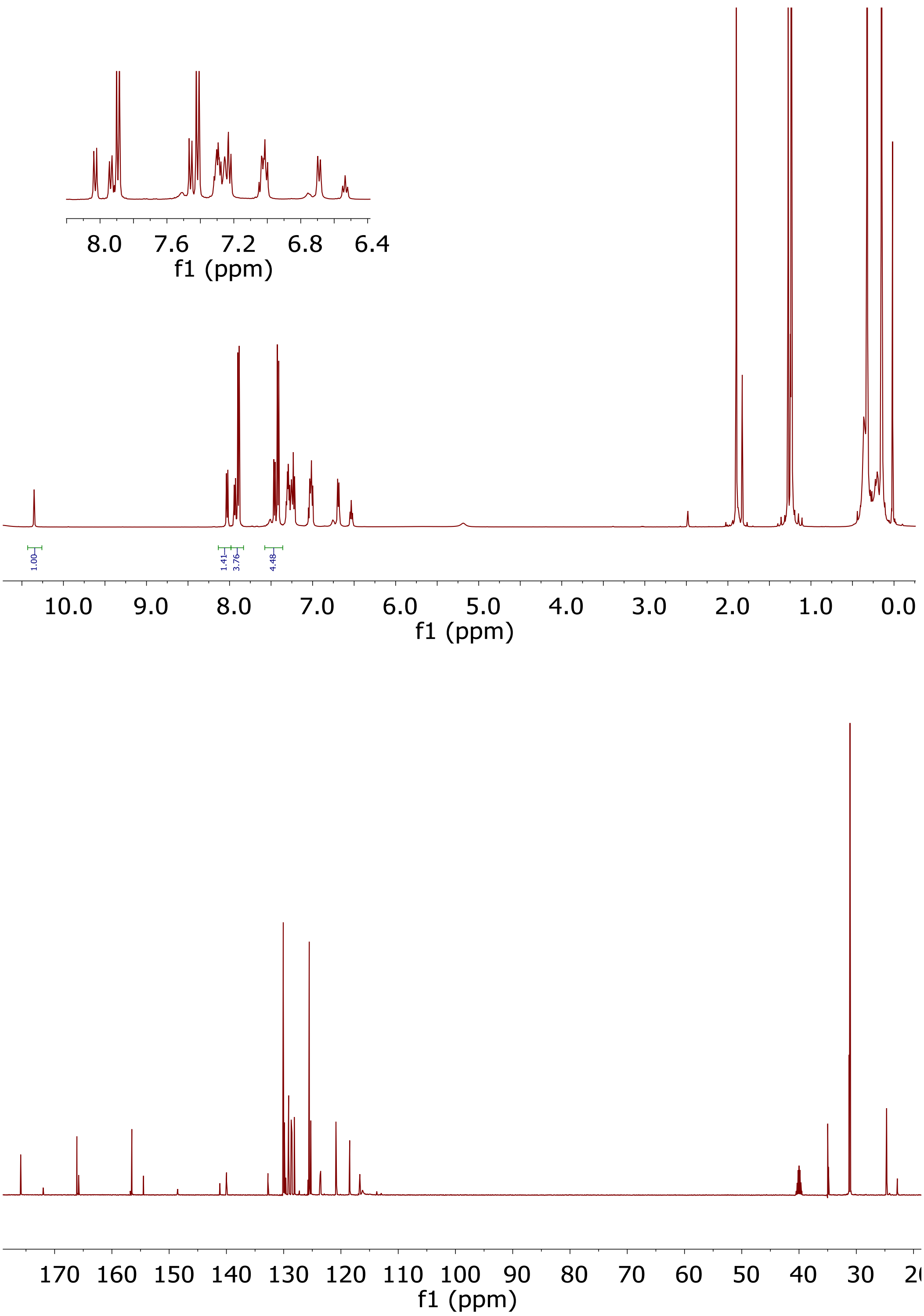

Figure S2. Reaction of silylated aniline (Figure S1) with 4-(*tert*-butyl)benzoyl chloride in DMSO- d_6. Top) ^{1}H-NMR spectrum; bottom) ^{13}C{^{1}H}-NMR spectrum.

b) In a second step, the silylation of the amide group of four benzanilide models (Scheme S2) was attempted in order to determine which isomer is the predominant one (Figure S3).

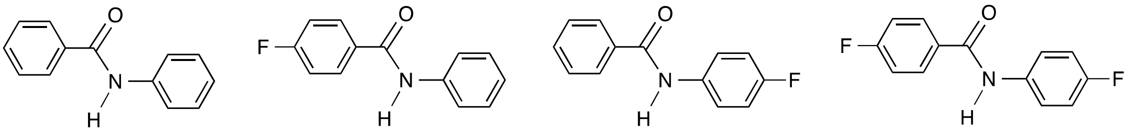

1a **1b** **1c** **1d**

Scheme S2. Benzanilide models

Benzanilide (*trans*-N-phenylbenzamide) **1a** and its *para*-fluoroderivatives **1b-1d** (Scheme S2) could be silylated in high yield, as estimated by NMR, on using 3 equivalents of a very reactive silylation reactant, N,O-bis(trimethylsilyl)acetamide (BSA), per mol of benzamide in N,N-dimethylacetamide (DMAc). In all cases, reaction mixtures of silylated benzamides were observed (Scheme S3) by ^{13}C and ^{1}H NMR, but it was not possible to discern the possible silylated amides (Scheme 2). However, when the reaction mixtures of the fluorinated benzamides were analyzed by ^{19}F NMR (using ^{19}F-^{19}F-EXSY (EXchange SpectroscopY)), their unambiguous characterization could be established. Thus, these reaction mixtures were composed of the O-silyl imidates isomers (*trans* (**2**) and *cis* (**3**), respectively) along with the N-silyl amide (**4**), which are exchanged with each other as will be demonstrated below. This exchange involving isomer **4**, had not been detected before[2], and suggests the participation of the silylating reagent in the amide modification process. To justify this fact, an experimental process, where a flame-sealed coaxial capillary containing acetone-d_6 to block the deuterium signal, was designed for the silylation of 4-fluoro-N-(4-fluorophenyl)benzamide (**1d**).

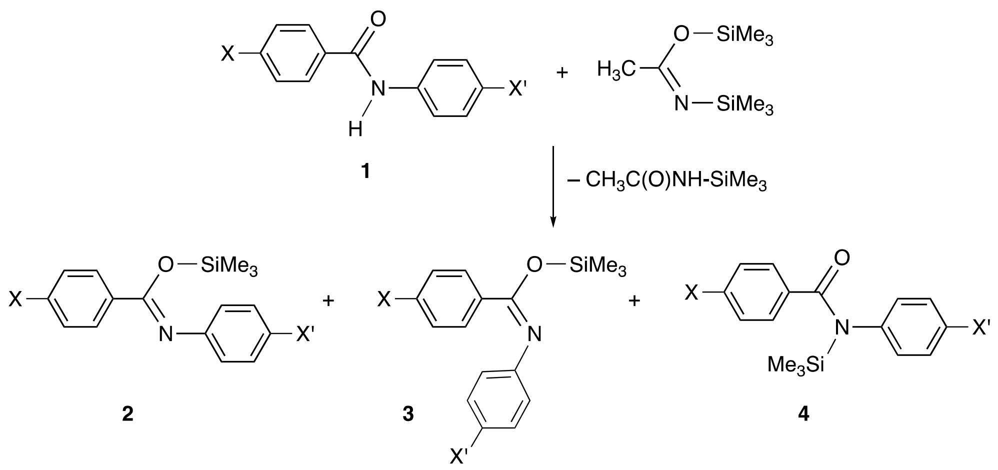

Scheme S3. Silylation of benzanilides

The quantitative ^{19}F NMR spectrum at 283 K of **1d** treated with BSA in DMAc showed two sharp multiplets in very small proportion, due to the fluorine atoms of the starting material **1d**, and five slightly broad signals that correspond to the fluorine atoms of **2d**, **3d** and **4d**. (see Figure S3). The assignment of these signals to the different compounds was performed on the basis of the experiments described below.

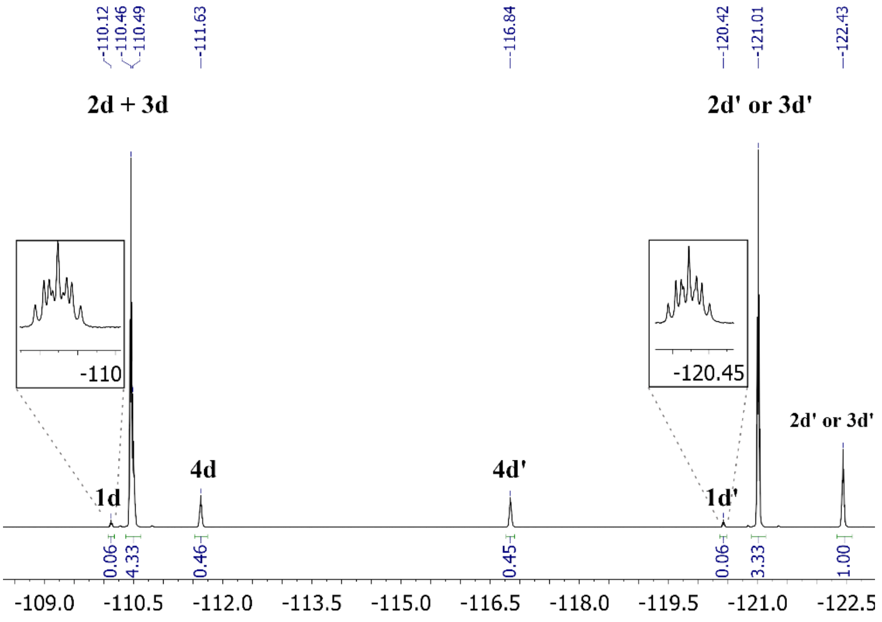

Figure S3. ^{19}F NMR (470 MHz, acetone-d_6 cap.) spectrum at 283 K from the silylation reaction of **1d** with BSA in DMAc. Note: the quotation mark (') depicts the fluorine atom on the right in the structures shown in Scheme 3.

The molar ratio found for the two major compounds, which accounts for the 90% of the integrated signals (the two O-silyl imidate isomers), was 69/21 while the remaining 10% was ascribed to the N-silyl amide **4d** and the starting material **1d** (9/1). Similar molar ratios were found in the ^{19}F NMR experiment performed at 298 K (Figure S4), while the width of the signals was more pronounced.

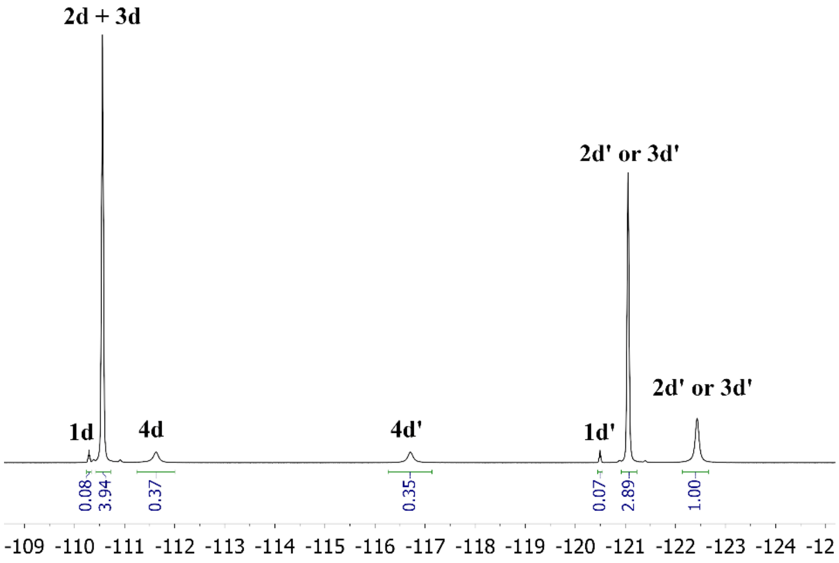

Figure S4. ^{19}F NMR (470 MHz, acetone-d_6 cap.) spectrum at 298 K from the silylation reaction of **1d** with BSA in DMAc.

As expected from the appearance of the signals, the two-dimensional ^{19}F-^{19}F EXSY experiment at 283 K (Figure S5) showed that **1d** does not participate in the exchange between species (**2d**, **3d,** and **4d**) at this temperature and that the two minor species (excluding **1d**) exchanged more rapidly with each other than with the third species (the major isomer). The existence of these exchanges reveals that in the reaction mixture the quantities observed for **2d-4d** corresponded to those of the thermodynamic equilibrium between species.

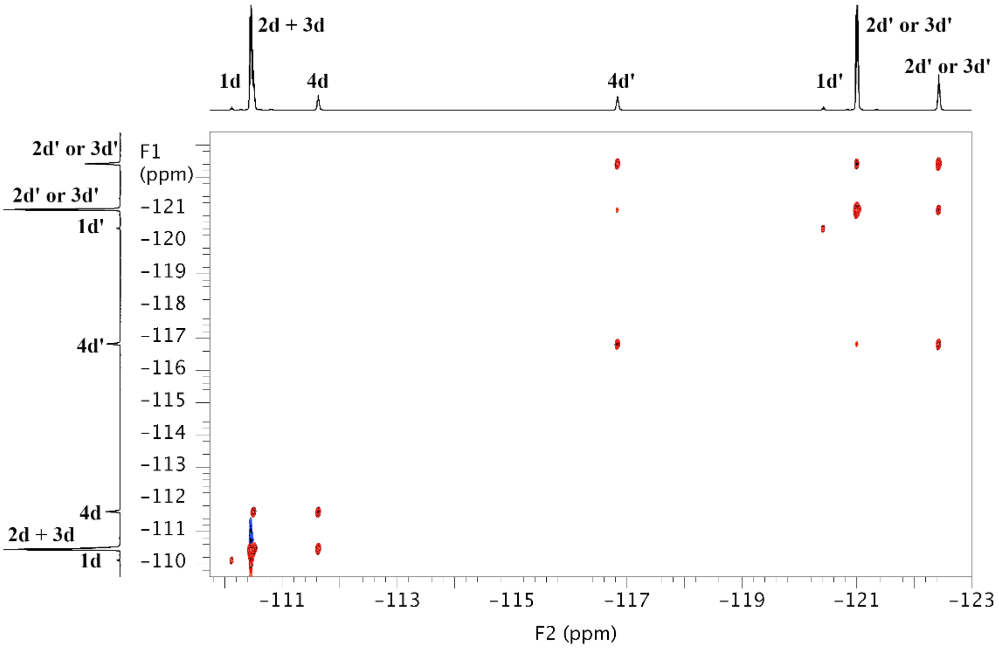

Figure S5. ^{19}F-^{19}F EXSY (470 MHz, acetone-d_6 cap.) spectrum at 283 K from the silylation reaction of **1d** with BSA in DMAc.

Tanabe et al[2] reported the existence of the two unassigned *trans/cis* O-silyl imidate isomers (they observed two signals in the 233 K $^{29}Si\{^1H\}$ NMR spectrum at 23.25 and 23.10 ppm) for the product isolated on the silylation of benzanilide with *tert*-butyldimethylsilyl chloride in the presence of NaH. Our experiments are in good agreement with these data and two broad signals were observed in the 283 K $^{29}Si\{^1H\}$ for the silylation of **1d** at 20.92 and 20.88 ppm which should correspond to **2d**/**3d** (see Figure S6).

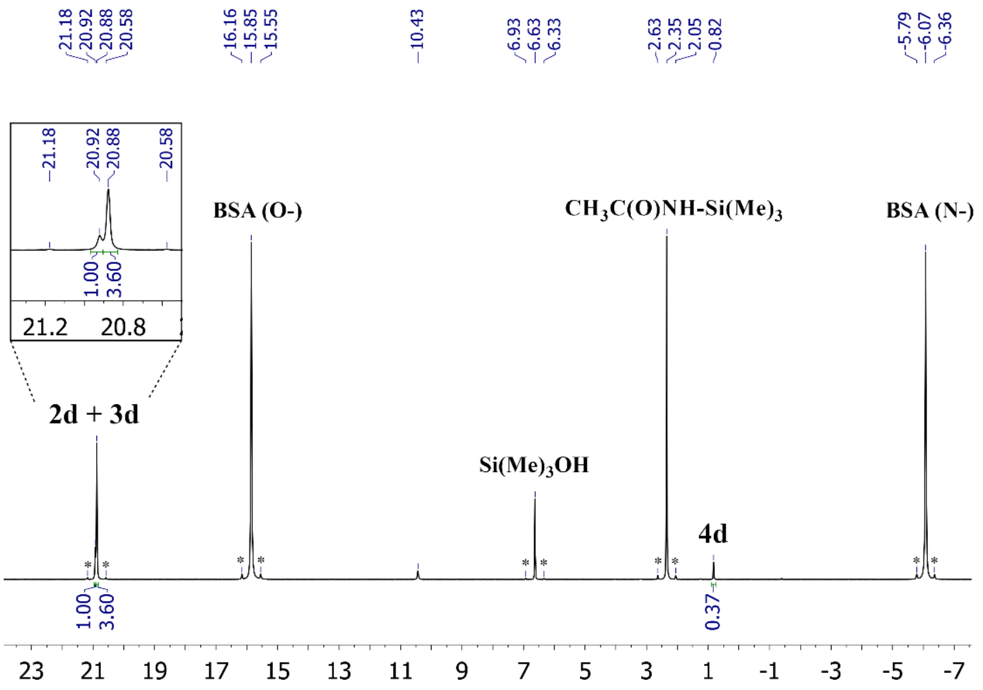

Figure S6. Quantitative $^{29}Si\{^1H\}$ NMR (99 MHz, acetone-d_6 cap.) spectrum at 283 K from the silylation reaction of **1d** with BSA in DMAc showing the different types of $SiMe_3$ presents and their corresponding ratio. Note: the ^{29}Si resonances shown with black asterisks correspond to satellites of ^{13}C.

In addition to the signals assigned for BSA (the by-product $CH_3C(O)NH\text{-}SiMe_3$ and the hydrolysis product $SiMe_3OH$), a sharp singlet at 0.82 ppm was observed in the NMR spectrum of the mixture reaction in the NMR tube. This new resonance peak was assigned to the N-silyl amide **4d** (not detected by Tanabe[2]) where the integration of this peak obtained in the quantitative $^{29}Si\{^1H\}$ experiment was similar to that obtained in the quantitative ^{19}F NMR spectrum (Figure S3, signals **4d** and **4d'**). Considering the complete chemical shift assignment of the ^{29}Si, a $^1H\text{-}^{29}Si$ HMBC experiment was performed to determine which protons are correlated with each silicon nucleus (see Figure S7).

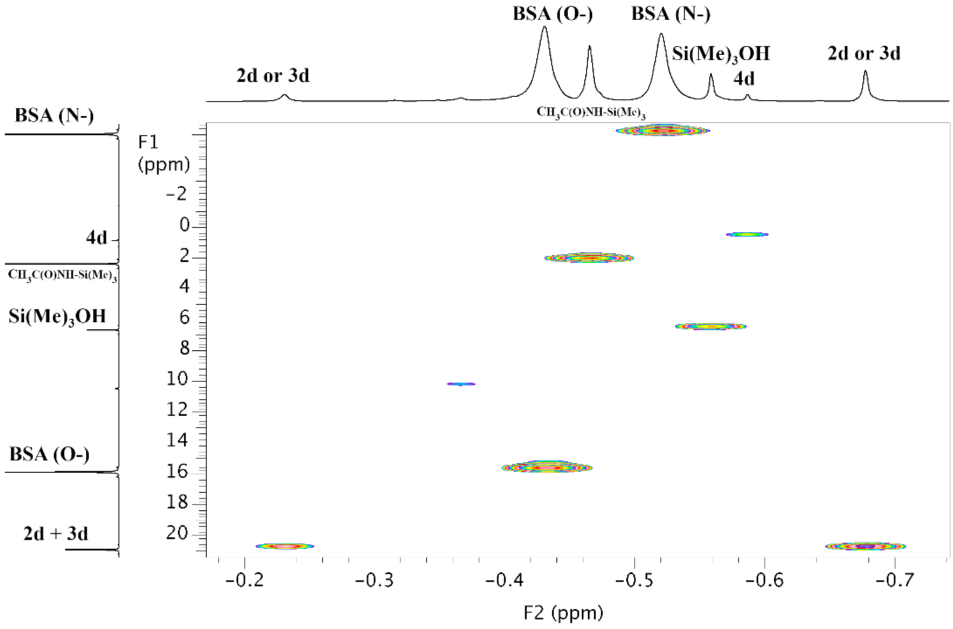

Figure S7. ^{1}H–^{29}Si HMBC (500 MHz, acetone-d_6 cap.) spectrum at 283 K from the silylation reaction of **1d** with BSA in DMAc showing the different cross-peaks of $SiMe_3$ region.

Despite the low natural abundance of ^{15}N nuclei, this atom is a good candidate to determine the existence of silylated products in an NMR tube because ^{15}N atoms present different environments. Thus, to confirm the presence of the **4d** N-silylamide, a ^{1}H-^{15}N HMBC spectrum was performed at 283 K (Figure S8). The most informative cross-peak observed in this 2D spectrum optimized for $^{3}J_{1H\text{-}15N}$ correlations presented a value of –355.20 ppm in the ^{15}N scale (F1) which correlated with a single proton at –0.59 ppm in the $SiMe_3$ region (F2). This result confirmed the presence of **4d** in solution because it is the only methylsilyl group, present in the isomer mixture, able to correlate with a ^{15}N center. Obviously, this correlation is not possible for the **2d** and **3d** O-silyl imidates.

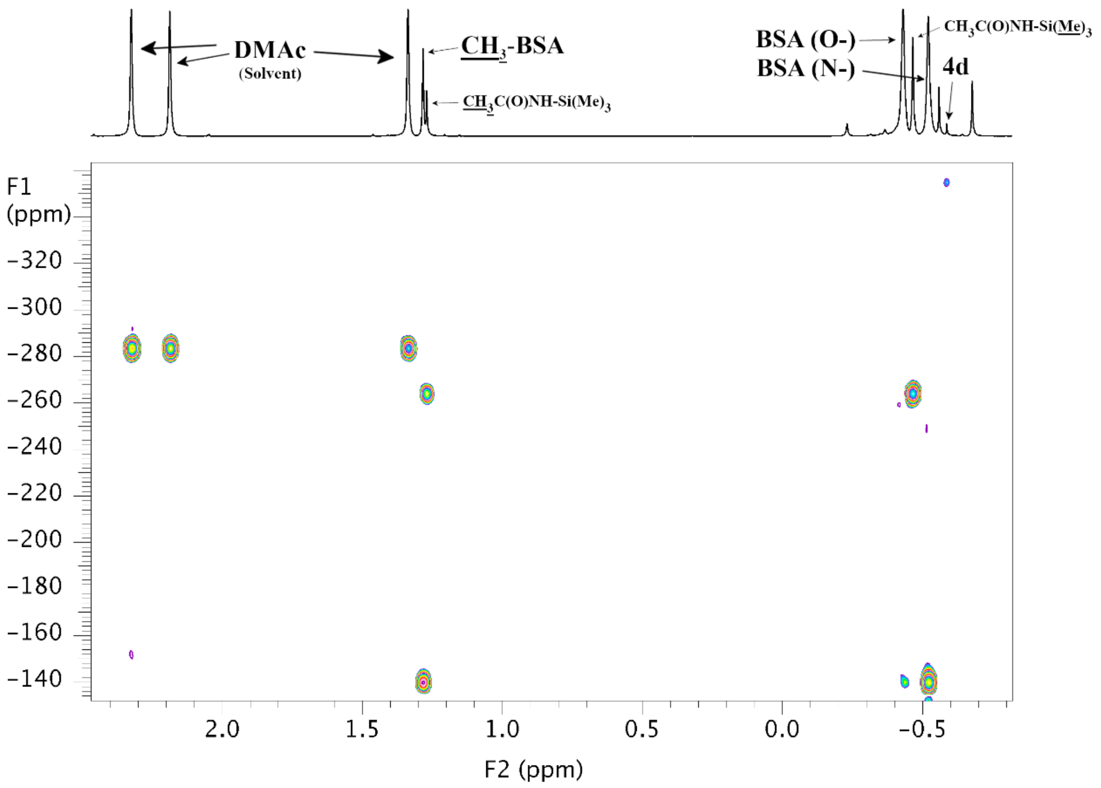

Figure S8. ^{1}H–^{15}N HMBC (500 MHz, acetone-d_6 cap.) spectrum at 283 K from the silylation reaction of **1d** with BSA in DMAc showing the different chemicals shifts of ^{15}N found.

In the $^{19}F\{^{1}H\}$–^{15}N HMBC spectrum, optimized for the long-range $^{5}J_{19F\text{-}15N}$ coupling, two different ^{15}N chemical shifts (NMR peaks at –126.24 and –126.85 ppm, which can be associated with the *cis* and *trans* O-silyl imidates) were observed (Figure S9). Similar ^{15}N chemical shifts were reported for Tanabe employing a ^{15}N-enriched sample[2]. The high chemical shift dispersion of more than 200 ppm observed for the ^{15}N centers in **4d** compared to **2d** and **3d** can be attributed to the different electron density bearing the nitrogen nucleus.

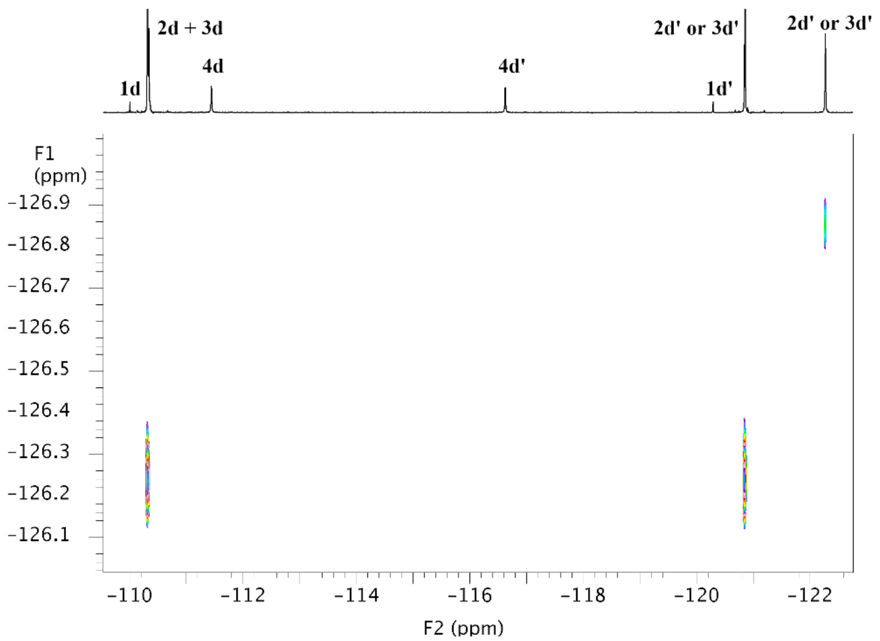

Figure S9. $^{19}F\{^{1}H\}–^{15}N$ HMBC (470 MHz, acetone-d_6 cap.) spectrum at 283 K from the silylation reaction of **1d** with BSA in DMAc showing the two different chemicals shifts of ^{15}N *trans/cis* O-silyl imidates isomers. The experiment was optimized for $^{5}J_{19F-15N}$ = 5 Hz.

Finally, it should be remarked that the molar ratios observed for the silylated species **2d**/**3d** and **4d** were consistent with the ^{19}F, ^{1}H, $^{13}C\{^{1}H\}$ and $^{29}Si\{^{1}H\}$ recorded quantitative NMR spectra (see Figures S3, S10, S11, S6).

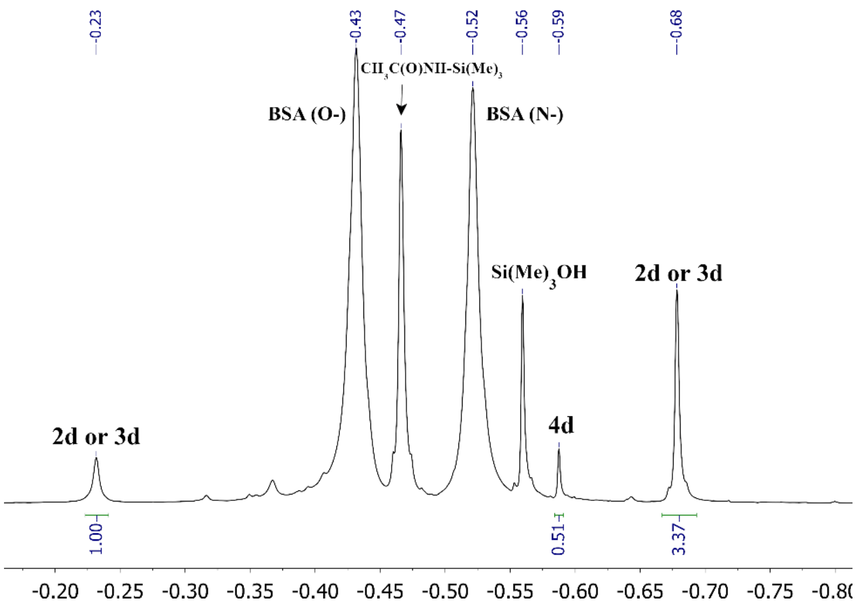

Figure S10. ^{1}H NMR (500 MHz, acetone-d_6 cap.) spectrum at 283 K from the silylation reaction of **1d** with BSA in DMAc showing the different types of $SiMe_3$ presents and their corresponding ratio.

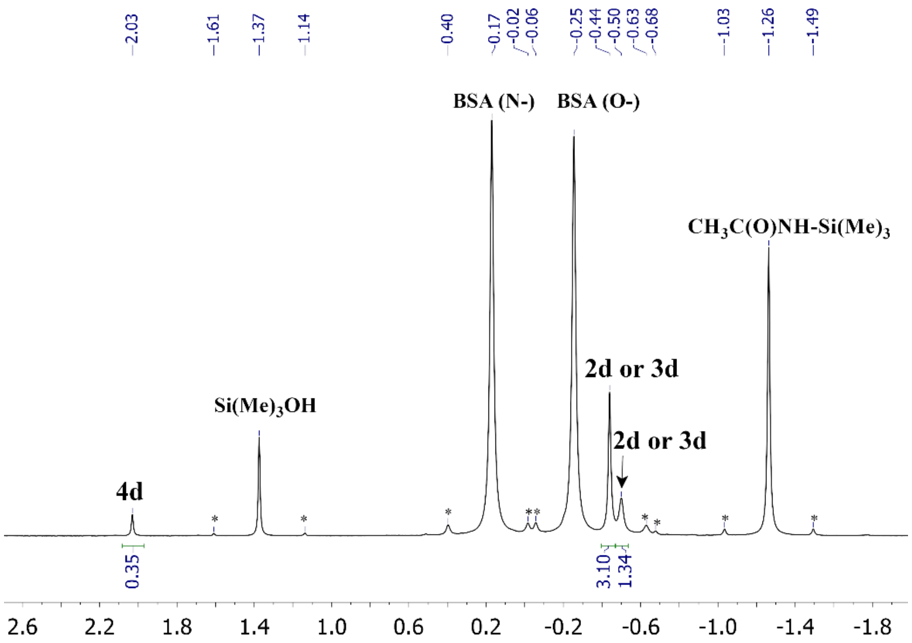

Figure S11. Quantitative $^{13}C\{^{1}H\}$ NMR (125 MHz, acetone-d_6 cap.) spectrum at 283 K from the silylation reaction of **1d** with BSA in DMAc showing the different types of $SiMe_3$ presents and their corresponding ratio. Note: the ^{13}C resonances shown with black asterisks correspond to satellites of ^{29}Si. One satellite is overlapped with the **2d** or **3d** resonance resulting in a total of 1.34 integral value.

Figure S12 shows the ^{1}H–^{13}C HSQC spectrum at 283 K of the silylation reaction of **1d** with BSA in DMAc for $SiMe_3$ region.

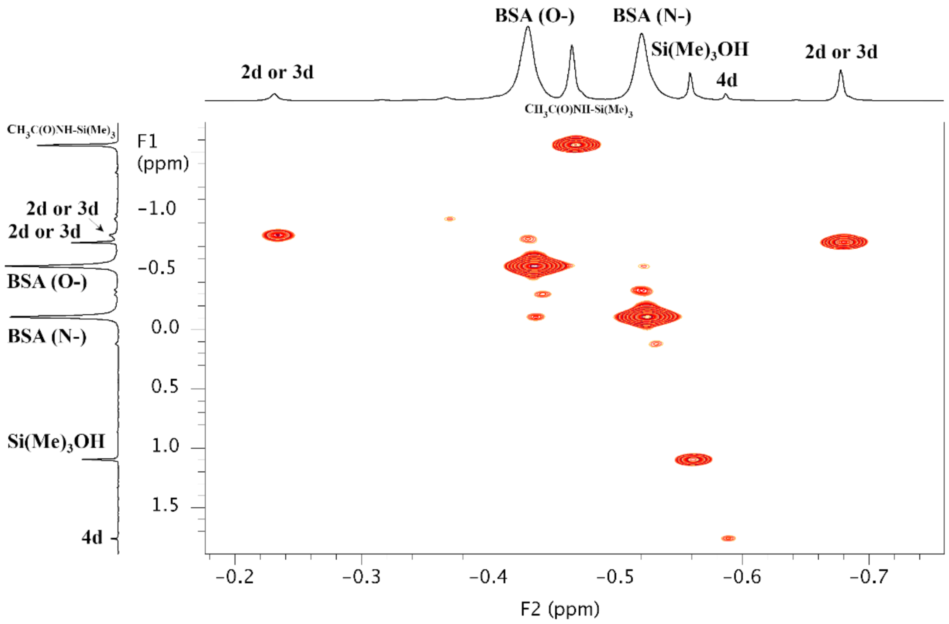

Figure S12. ^{1}H–^{13}C HSQC (500 MHz, acetone-d_6 cap.) spectrum at 283 K from the silylation reaction of **1d** with BSA in DMAc showing the different cross-peaks of $SiMe_3$ region.

In conclusion, this NMR study has allowed us to determine the ability of the amide group of benzanilides to be silylated, using a strong silylating agent (BSA). Also, it has been unequivocally observed that the predominant silylated benzanilide isomers are silyl imidates **2** and **3**.

S5. Molecular simulation study

After the NMR experimental study, it was considered interesting to carry out a DFT quantum mechanical study to determine the electronic energies of the entities **2**, **3**, and **4** (Scheme S3) derived from the benzanilides depicted in Scheme S2.

The energies of these three isomers, both in the gas phase and in DMSO, will be used to validate the molecular simulation method with the data obtained by NMR.

For the benzanilide without fluorine atoms, the Gibbs energy, at 298K, was also determined both in the gas phase and DMSO, since this structure will be used to build the macromolecular chain of PPTA and silylated PPTAs as discussed below.

Finally, a comparison between the macromolecular chains of PPTA and silylated PPTAs will be carried out using the molecular modeling package MaterialsStudio to determine whether the differences between their conformations could be used to justify the changes in molecular weights observed experimentally.

S5.1. Molecular simulation methods

Computer simulations were carried out by first drawing the molecules in GaussView 5[3] and then optimizing the structures at the AM1 level.[4] Subsequently, geometric features and electronic and Gibbs (at 298K) energies of the optimized geometries were determined by density functional theory (DFT) without any geometrical constraint (use of Opt and Force keywords for starting molecules) using the Becke's three-parameter hybrid function with the 6-31G(d,p) basis set (B3LYP/6-31G(d,p))[5,6] using the Gaussian 09 program.[7,8]. All the optimized structures were also calculated by considering solvation effects, using the Polarizable Continuum Model (PCM) using the integral equation formalism variant (IEFPCM)[9], considering DMSO as the solvent.

Molecular depictions of molecules were created using the Arguslab 4.01 freeware program[10] and the Gaussview 5 program.[3]

The optimized molecules (silylated and non-silylated ones) were used as monomeric units to produce polymeric chains (10 structural units) that were constructed by using the Polymer Builder

module of MaterialsStudio[11], with a random torsion angle between the units. The obtained geometry of the macromolecular chains was optimized by the Forcite module of Materials Studio by doing, sequentially, the following tasks; a) geometry optimization (Compass forcefield, using the Smart algorithm, with quality medium), b) dynamics (using the Ensemble NVT at 400K for a total simulation of 20 ps), and c) geometry optimization (Compass forcefield using the Smart algorithm, with quality fine).

S5.2Molecular simulation discussion

A DFT (B3LYP/6-31G(d,p)) quantum-mechanic study was carried out for the models depicted in Scheme S4.

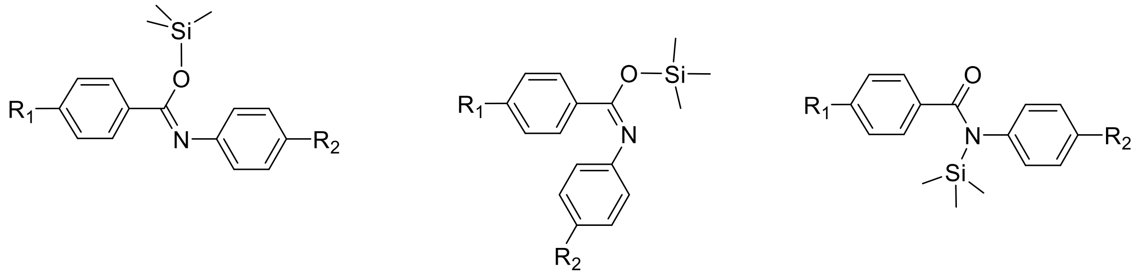

**2a**: $R_1 = R_2 = H$	**3a**: $R_1 = R_2 = H$	**4a**: $R_1 = R_2 = H$
**2b**: $R_1 = F$; $R_2 = H$	**3b**: $R_1 = F$; $R_2 = H$	**4b**: $R_1 = F$; $R_2 = H$
**2c**: $R_1 = H$; $R_2 = F$	**3c**: $R_1 = H$; $R_2 = F$	**4c**: $R_1 = H$; $R_2 = F$
**2d**: $R_1 = R_2 = F$	**3d**: $R_1 = R_2 = F$	**4d**: $R_1 = R_2 = F$

Scheme S4. Isomers of benzanilides

Table S4 shows the electronic energies of the benzanilide model isomers. It can be seen that the lowest electronic energy values (both in the gas phase and in DMSO) corresponded to O-silyl imidates **2** (*trans*) and **3** (*cis*) (Scheme S4). The energetic differences between these O-silyl imidates were around 1 kcal/mol, showing the lowest energetic value for O-silyl imidates (**3**). The energy of N-silylamides (4) were much higher (> 4 kcal/mol) than that of the O-silyl imidates (2 and 3). This result was in full agreement with the NMR study, where it was observed that in solution the number of silylated species followed the order: *cis* O-silyl imidates **3** > *trans* O-silyl imidates **2** >> N-silyl amides **4**.

For the benzanilide without fluorine atoms, the Force Gaussian keyword was used to determine the Gibbs energy at 298 K for species **2a**, **3a**, and **4a**.

The Gibbs energy results presented in Table S5 and for O-silyl imidates **2a** and **3a**, were very similar to those obtained from the electronic energies. For N-silyl amide **4a**, it was observed that the Gibbs energy differences between **4a** and species **2a** and **3a** were somewhat larger (> 6 kcal/mol).

Table S4. Electronic energy values of benzanilide models (Scheme S4)

**Model**	**Gas phase**		**DMSO**		**Gas phase**	**DMSO**
	**Electronic energy (Ee)**		**Electronic energy (Ee)**		**ΔEe**	**ΔEe**
	Hartree	kcal/mol	Hartree	kcal/mol	kcal/mol	kcal/mol
**2a**	-1040.724	-653064.10	-1040.730	-653067.96	1.12	0.84
**3a**	-1040.726	-653065.22	-1040.732	-653068.79	0	0
**4a**	-1040.713	-653057.25	-1040.723	-653063.58	7.97	5.22
**2b**	-1139.956	-715333.21	-1139.962	-715336.96	1.06	0.77
**3b**	-1139.958	-715334.27	-1139.964	-715337.73	0	0
**4b**	-1139.945	-715326.15	-1139.955	-715332.39	7.06	5.34
**2c**	-1139.955	-715332.59	-1139.962	-715336.46	0.92	0.69
**3c**	-1139.957	-715333.51	-1139.963	-715337.15	0	0
**4c**	-1139.945	-715325.88	-1139.955	-715332.14	7.63	5.02
**2d**	-1239.188	-777601.68	-1239.194	-777605.49	0.89	0.64
**3d**	-1239.189	-777602.56	-1239.195	-777606.13	0	0
**4d**	-1239.177	-777594.75	-1239.186	-777600.94	7.81	5.18

∗ΔEe refers to the relative electronic energies, calculated by subtracting the Ee of each isomer from the Ee of the isomer with the lowest electronic energy.

Table S5. Gibbs energy values (298 K) of benzanilide models **2a**, **3a**, and **4a** (Scheme S4)

**Model**	**Gas phase**		**DMSO**		**Gas phase**	**DMSO**
	**Gibbs energy at 298 K (Eg)**		**Gibbs energy at 298 K (Eg)**		**ΔEg**	**ΔEg**
	Hartree	kcal/mol	Hartree	kcal/mol	kcal/mol	kcal/mol
**2a**	-1040.465	-652901.15	-1040.472	-652905.6	1.16	0.82
**3a**	-1040.466	-652902.31	-1040.473	-652906.4	0	0
**4a**	-1040.452	-652892.94	-1040.462	-652899.6	9.38	6.82

The DFT (B3LYP/6-31G(d,p)) molecular structures of benzanilide and **2a**, **3a** and **4a** silylated benzanilides are depicted in Figure S13 and S14

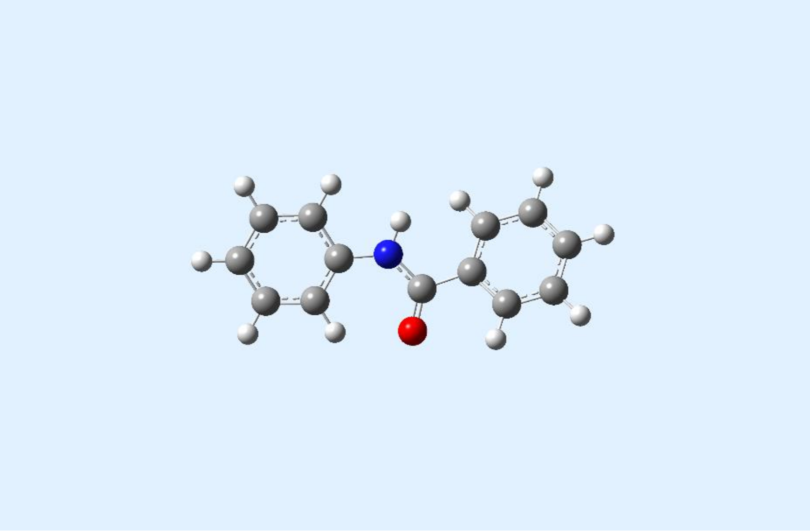

Figure S13. DFT Molecular modeling of benzanilide

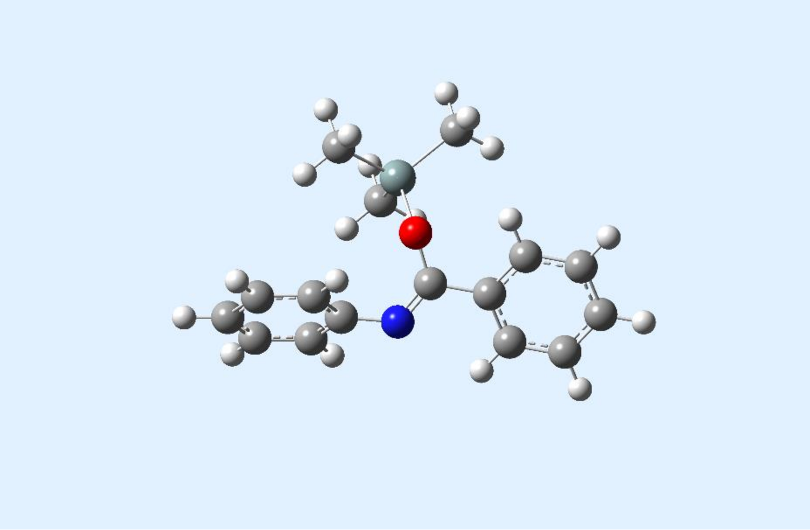

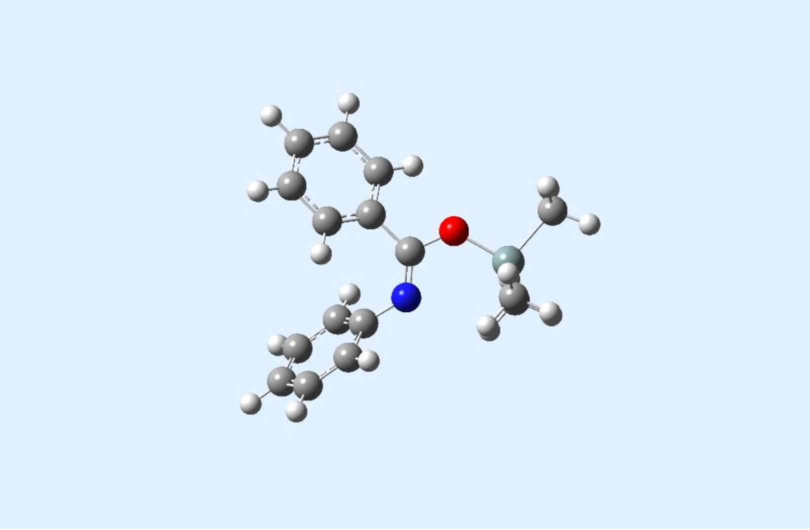

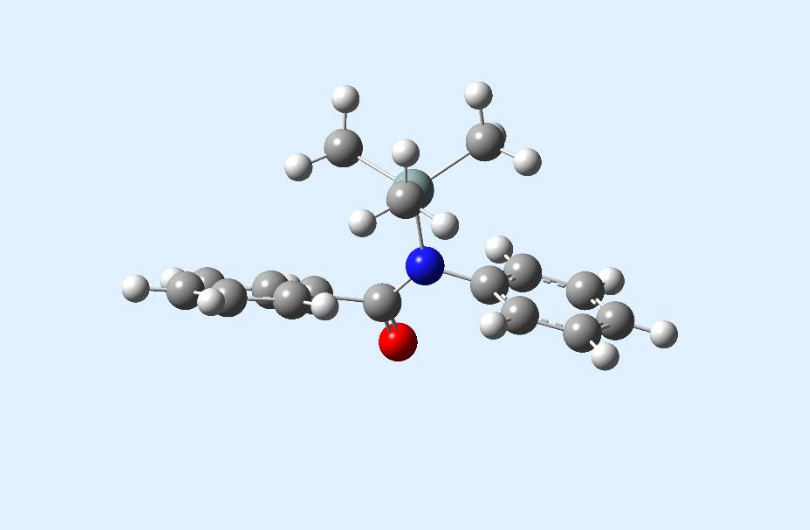

Figure S14. DFT Molecular modeling molecules **2a** (top figure), **3a** (middle figure), and **4a** (down figure) (Scheme S4)

With these optimized molecules, monomer structural units were created to obtain by Polymer Builder (Materials Studio) macromolecular chains of the three silylated PPTAs and PPTA.

The **PPTA** with 10 monomeric units is shown in Figure S15. It can be seen that this structure is quite rod-like.

Molecular modeling of the three silylated PPTAs showed the following facts: the *trans* O-silyl PPTA structure was completely rod-shaped, as observed in Figure S16, the *N*-silyl PPTA (Figure S17) was less rod-shaped than PPTA or *trans* O-silyl PPTA. However, *cis* O-silyl PPTA (Figure S18), due to the conformation of the O-silyl amide group, produced a highly coiled and entangled PPTA. This entangled conformation is very rigid and, therefore, there must be significant steric effects that could hinder the growth of the macromolecular chain. However, this anomalous conformation, if present, would completely modify the PPTA structure, separating the chains, which would make the growing macromolecular chain more soluble.

To justify this idea, the modeling of a silylated PPTA chain was performed, where 90% of the amide moieties possess *trans* O-silyl groups, and the remaining 10% *cis* O-silyl groups. In Figure S19, it is observed that the presence of *these* cis O-silyl groups produces an important

conformational change in the rod-shaped characteristics of PPTA, such that the polymer should be more soluble in the reaction medium.

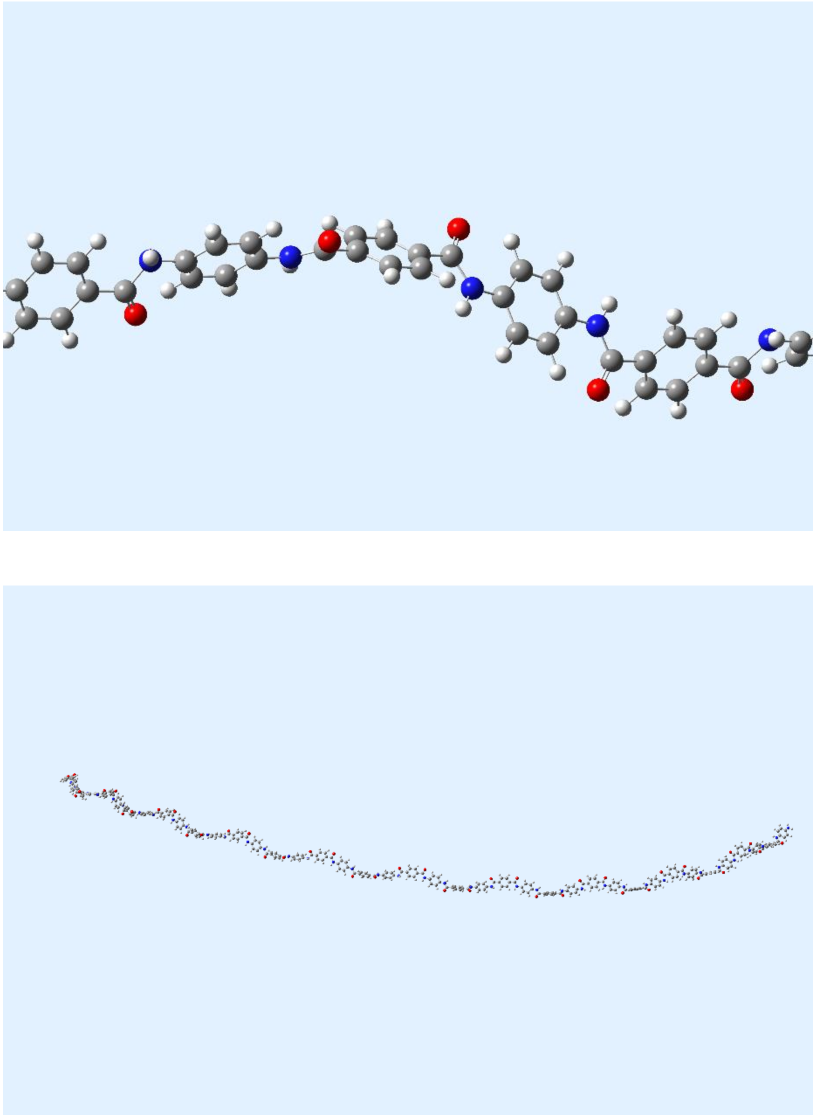

Figure S15. Molecular modeling of **PPTA** (bottom figure), enlargement (up figure)

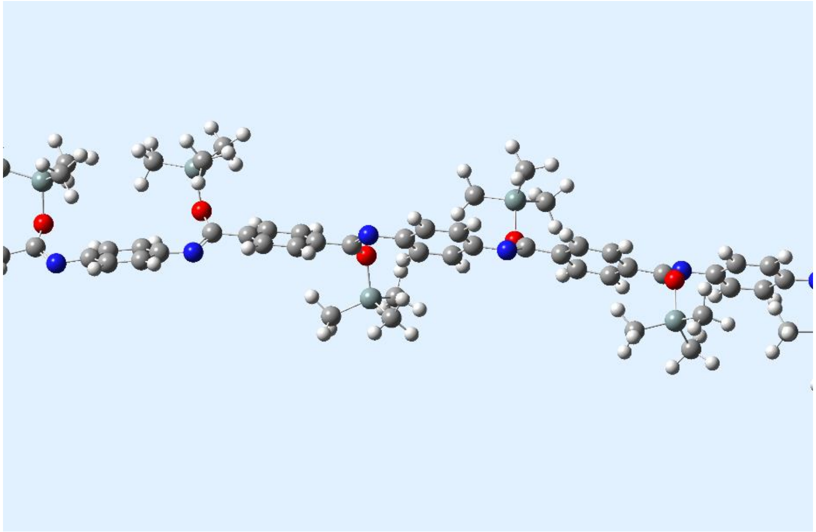

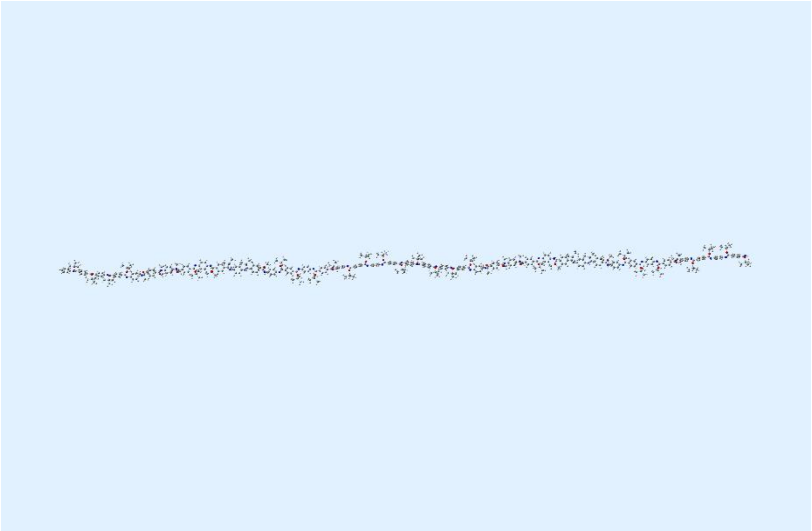

Figure S16. Molecular modeling of *trans* O-silylated **PPTA** (bottom figure), enlargement (up figure).

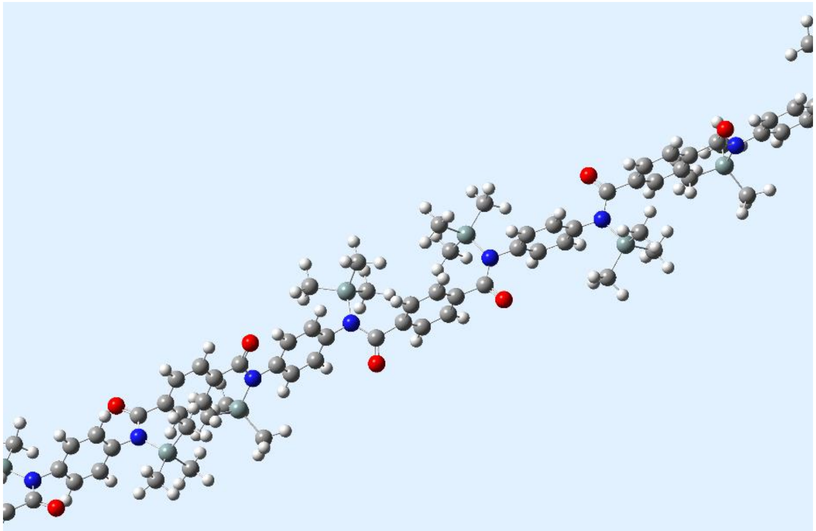

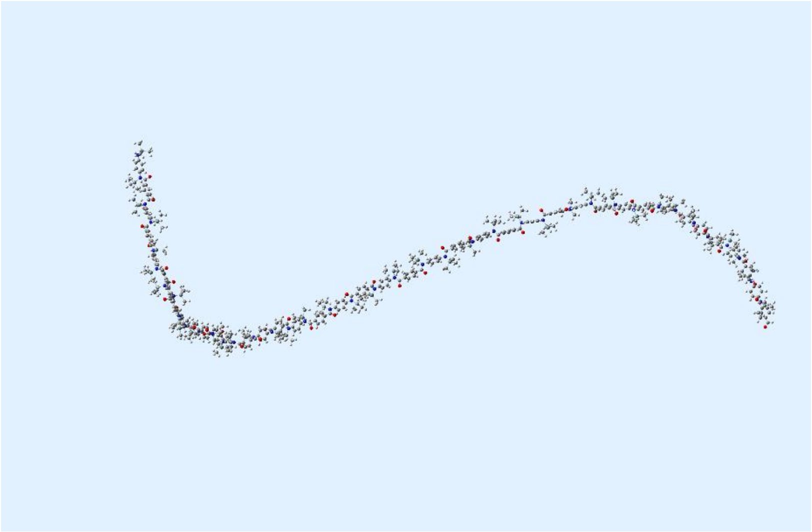

Figure S17. Molecular modeling of *N*-silylated **PPTA** (bottom figure), enlargement (up figure).

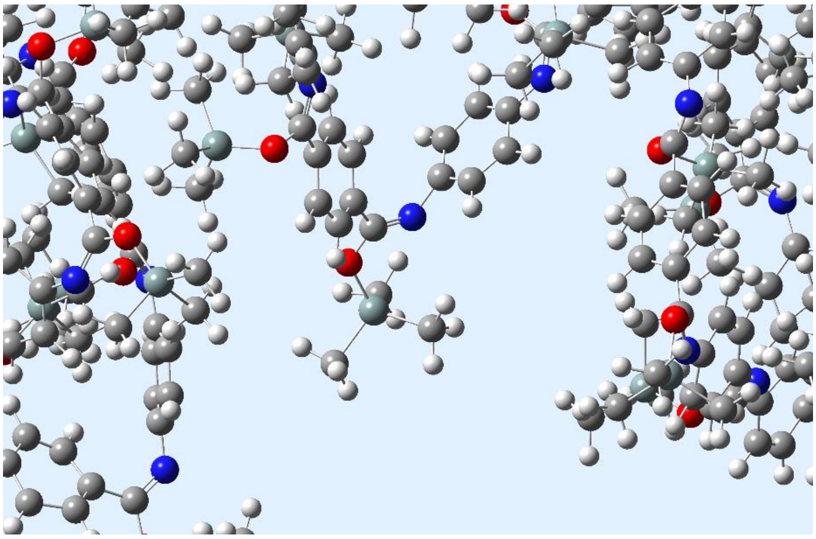

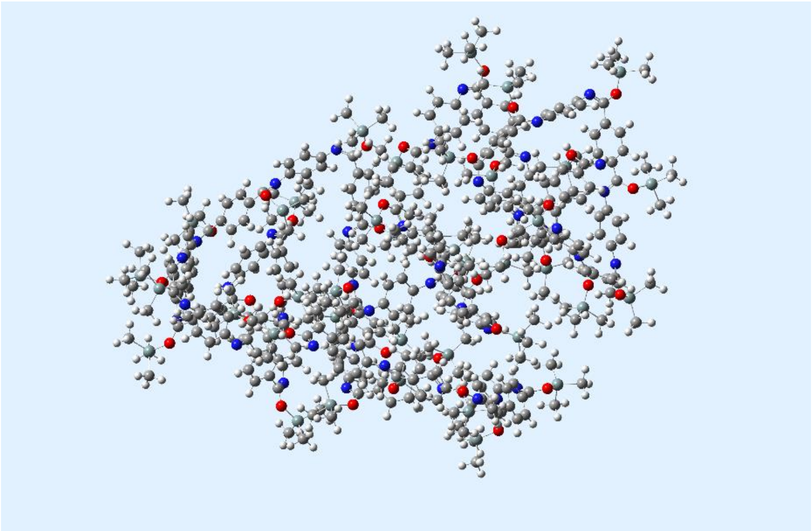

Figure S18. Molecular modeling of *cis* O-silylated **PPTA** (bottom figure), enlargement (up figure).

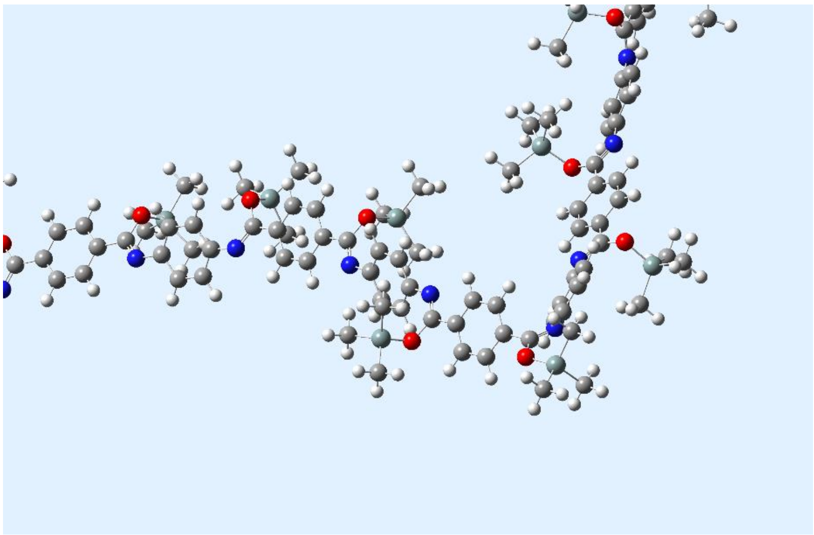

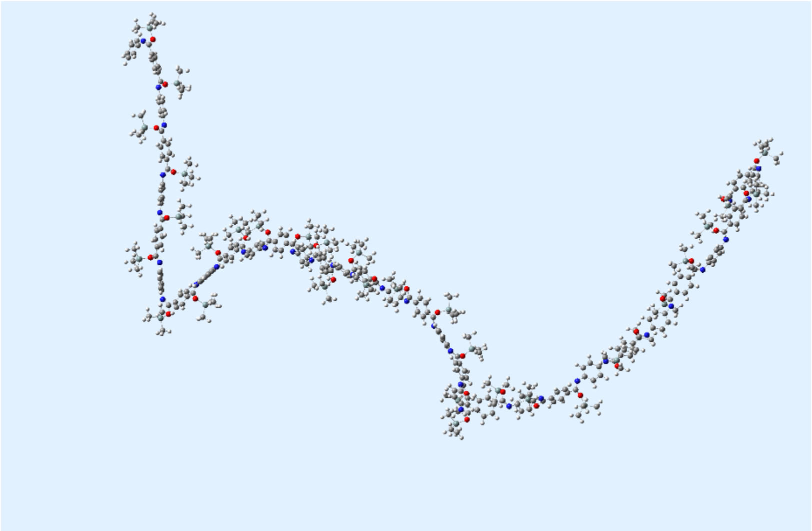

Figure S19. Molecular modeling of *cis* O-silylated/*trans* O-silylated (1/10) **PPTA** (bottom figure), enlargement (up figure).

S6. Molecular weight of PPTAs

Table S6. Inherent viscosities, and molecular weight, of PPTAs made in this work

PPTA batch	Inherent viscosity<br>dL/g	Molecular weight*<br>g/mol
1	3.15	23265
2	4.36	38581
3	6.72	75634
4	6.32	68746
5	6.58	73197
6	6.10	65059
7	9.4	127503

*Molecular weight of PPTAs was calculated[12,13] using the relationship $Mw=3902.4\eta^{1.556}$.

S7. Additional information

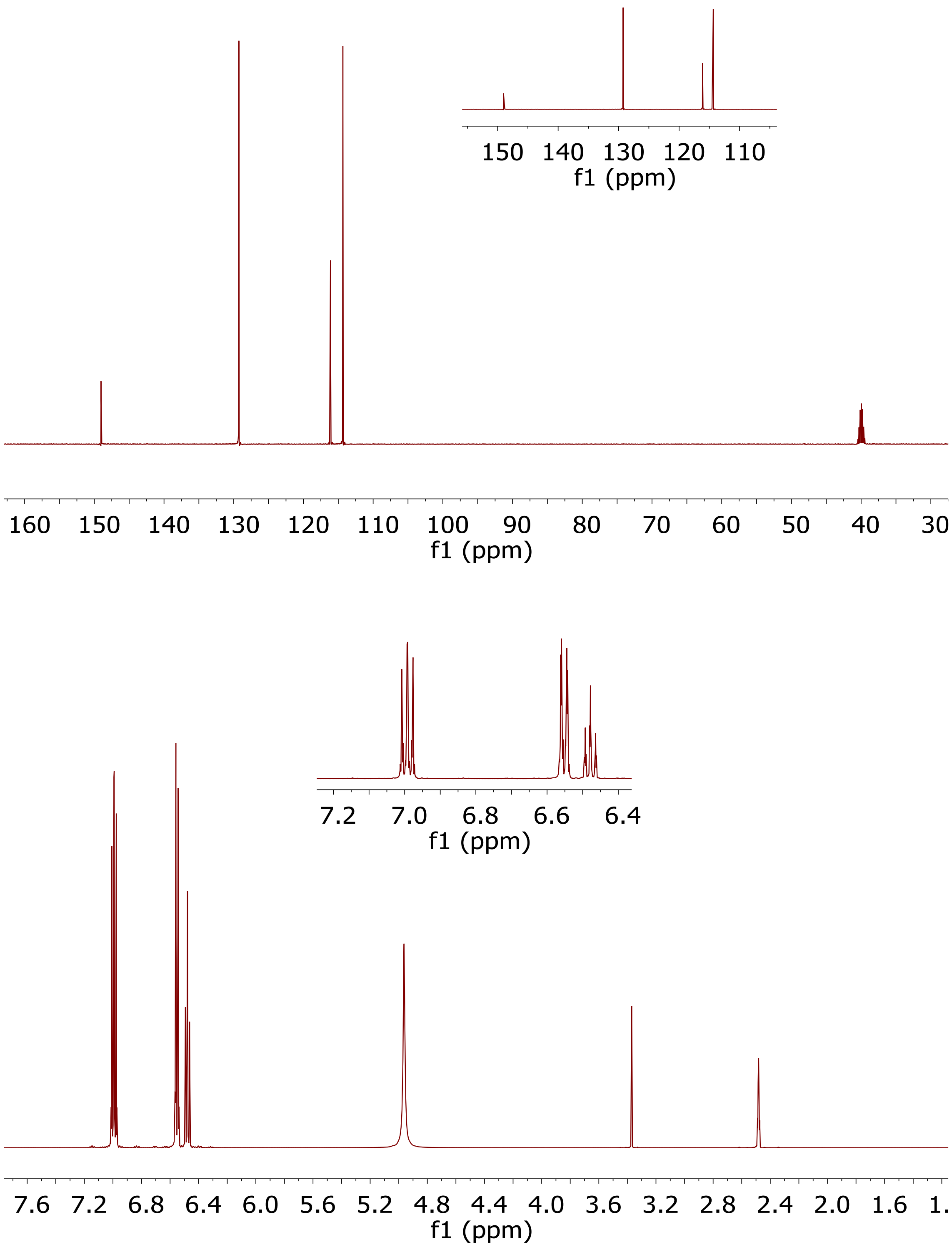

Figure S20. NMR of aniline, top (^{13}C-NMR), bottom (^{1}H-NMR)

References

1. Russo, S.; Mariani, A.; Ignatov, V.N.; Ponomarev, I.I. High-Molecular-Weight Aromatic Polyamides by Direct Polycondensation. *Macromolecules* **1993**, *26*, 4984–4985, doi:10.1021/ma00070a042.
2. Misaki, T.; Kurihara, M.; Tanabe, Y. Si-BEZA – Catalytic Pyridinium Triflate: A Mild and Powerful Agent for the Silylation of Alcohols. *Chem. Commun.* **2001**, *1*, 2478–2479, doi:10.1039/b107447b.
3. Dennington, R.; Keith, T.; Millam, J. GaussView, Version 5. *Semichem Inc. , Shawnee Mission. KS* 2009.
4. Dewar, M.J.S.; Zoebisch, E.G.; Healy, E.F.; Stewart, J.J.P. Development and Use of Quantum Mechanical Molecular Models. 76. AM1: A New General Purpose Quantum Mechanical Molecular Model. *J. Am. Chem. Soc.* **1985**, *107*, 3902–3909, doi:10.1021/ja00299a024.
5. Zhang, I.Y.; Wu, J.; Xu, X. Extending the Reliability and Applicability of B3LYP. *Chem. Commun. (Camb).* **2010**, *46*, 3057–3070, doi:10.1039/c000677g.
6. Foresman, J.B.; Frisch, A. *Gaussian Inc.* 2015,.
7. Jensen, J.O. Vibrational Frequencies and Structural Determination of Adamantane. *Spectrochim. Acta Part A Mol. Biomol. Spectrosc.* **2004**, *60*, 1895–1905, doi:10.1016/j.saa.2003.09.024.
8. Frisch (Gaussian, Inc.), Michael J. Trucks (Gaussian, Inc.), G. W. Schlegel (Gaussian, Inc.), H. Bernhard (Gaussian, I.. Gaussian 09, Revision A.02. *Gaussian 09, Revis. A.02* 2009.
9. Tomasi, J.; Mennucci, B.; Cammi, R. Quantum Mechanical Continuum Solvation Models. *Chem. Rev.* **2005**, *105*, 2999–3094, doi:10.1021/cr9904009.
10. M. A. Thompson ArgusLab 4.0.1 .
11. BIOVIA, Materials Studio, 2017R2, San Diego. *Dassault Systèmes* 2017.

12. Yang, H.H. *Kevlar Aramid Fibre*; John Wiley & Sons Ltd, Baffins Lane, Chichester, West Sussex PO19 1UD, UK, 1993; ISBN 0471937657.

13. Yan, H.; Li, J.; Tian, W.; He, L.; Tuo, X.; Qiu, T. A New Approach to the Preparation of Poly(p-Phenylene Terephthalamide) Nanofibers. *RSC Adv.* **2016**, *6*, 26599–26605, doi:10.1039/c6ra01602b.
